# Cathelicidin is a “fire alarm”, generating protective NLRP3-dependent airway epithelial cell inflammatory responses during infection with *Pseudomonas aeruginosa*

**DOI:** 10.1371/journal.ppat.1007694

**Published:** 2019-04-12

**Authors:** Brian J. McHugh, Rongling Wang, Hsin-Ni Li, Paula E. Beaumont, Rebekah Kells, Holly Stevens, Lisa Young, Adriano G. Rossi, Robert D. Gray, Julia R. Dorin, Emily L. Gwyer Findlay, David Brough, Donald J. Davidson

**Affiliations:** 1 Centre for Inflammation Research at the University of Edinburgh, Queens’ Medical Research Institute, Edinburgh BioQuarter, Little France Crescent, Edinburgh, United Kingdom; 2 Division of Neuroscience and Experimental Psychology, School of Biological Sciences, Faculty of Biology, Medicine and Health, Manchester Academic Health Science Centre, University of Manchester, Manchester, United Kingdom; University of Pennsylvania, UNITED STATES

## Abstract

Pulmonary infections are a major global cause of morbidity, exacerbated by an increasing threat from antibiotic-resistant pathogens. In this context, therapeutic interventions aimed at protectively modulating host responses, to enhance defence against infection, take on ever greater significance. *Pseudomonas aeruginosa* is an important multidrug-resistant, opportunistic respiratory pathogen, the clearance of which can be enhanced *in vivo* by the innate immune modulatory properties of antimicrobial host defence peptides from the cathelicidin family, including human LL-37. Initially described primarily as bactericidal agents, cathelicidins are now recognised as multifunctional antimicrobial immunomodulators, modifying host responses to pathogens, but the key mechanisms involved in these protective functions are not yet defined. We demonstrate that *P*. *aeruginosa* infection of airway epithelial cells promotes extensive infected cell internalisation of LL-37, in a manner that is dependent upon epithelial cell interaction with live bacteria, but does not require bacterial Type 3 Secretion System (T3SS). Internalised LL-37 acts as a second signal to induce inflammasome activation in airway epithelial cells, which, in contrast to myeloid cells, are relatively unresponsive to *P*. *aeruginosa*. We demonstrate that this is mechanistically dependent upon cathepsin B release, and NLRP3-dependent activation of caspase 1. These result in LL-37-mediated release of IL-1β and IL-18 in a manner that is synergistic with *P*. *aeruginosa* infection, and can induce caspase 1-dependent death of infected epithelial cells, and promote neutrophil chemotaxis. We propose that cathelicidin can therefore act as a second signal, required by *P*. *aeruginosa* infected epithelial cells to promote an inflammasome-mediated altruistic cell death of infection-compromised epithelial cells and act as a “fire alarm” to enhance rapid escalation of protective inflammatory responses to an uncontrolled infection. Understanding this novel modulatory role for cathelicidins, has the potential to inform development of novel therapeutic strategies to antibiotic-resistant pathogens, harnessing innate immunity as a complementation or alternative to current interventions.

## Author summary

Lung infections are a common cause of death worldwide. As the threat of antibiotic-resistance becomes realised, new approaches are needed to treat disease-causing bacteria, such as multidrug-resistant *Pseudomonas aeruginosa*. Treatments that could enhance the body’s most effective natural defences can overcome antibiotic-resistance issues and/or complement existing therapies. Antimicrobial Host Defense Peptides, such as human LL-37, can kill microbes, but also have vital roles in clearing lung infections by modifying naturally-occurring defenses. Understanding these “immunomodulatory” activities is key to harnessing their potential. Airway lining cells have important barrier roles, but, if infected, these compromised cells must be removed and signal danger, to prevent harmful bacteria establishing a protected site and proliferating. We show that when *Pseudomonas aeruginosa* infects these cells, LL-37 can provide a second signal, acting like a fire alarm, instructing the compromised cells to signal the danger, to recruit host defence cells to the site, and to activate their own altruistic death. We demonstrate the mechanism of this process, from bacterial and host cell perspectives, occurring by activation of an intracellular sensing platform (the NLRP3 inflammasome) with a novel synergy between the infection and the impact of LL-37, with implications for innovative new approaches to treat multi-drug resistant infections.

## Introduction

Pulmonary diseases caused by bacterial or viral infections are a common cause of morbidity and account for 1 in 5 deaths in the UK [1]. There is an increasing global threat from antibiotic-resistant bacterial infections and newly emerging viral infections. In this context, therapeutic interventions aimed at protectively modulating host responses, to enhance defence against infection, take on greater significance as alternative or complementary future approaches. An important component of first line defences against such infections is the innate immune response of airway epithelial cells, which constitute the principal barrier first encountered by respiratory pathogens. These innate epithelial cell responses include the secretion of antimicrobial host defence peptides (HDP) [2, 3], and the release of chemokines and cytokines to help orchestrate the response of other immune effector cells. HDP are produced by innate immune effector cells in response to infection, damage and inflammation [4] Understanding their effect on epithelial cell responses to infection is important in elucidating the potential of HDP as targets for development of future antimicrobial interventions.

HDP are evolutionarily conserved short cationic peptides with direct and indirect antimicrobial activity, produced by a wide range of cells, including neutrophils, macrophages and epithelial cells. In addition to their well-characterised microbicidal activity against bacteria, viruses and fungi [4, 5], HDP can have a wide range of immunomodulatory and inflammomodulatory properties [4, 6]. The cathelicidin family of HDP, including the sole human cathelicidin LL-37/h-CAP18, has a particularly broad range of modulatory properties and has been shown to be critical to host defence against infections *in vivo* in a wide range of systems [7], including bacterial and viral pulmonary infections [8–10]. Cathelicidin can promote clearance of bacterial lung infection *in vivo* specifically by inflammomodulatory mechanisms, in the absence of susceptibility of the pathogen to direct peptide-mediated killing [8]. However, the mechanisms employed to alter the nature and/or efficacy of host responses to bacterial lung infection *in vivo* remain unclear. HDP, including cathelicidin, have been shown to modulate innate pattern recognition receptor (PRR) responses in host cells, including airway epithelial cells [11–13]. This raises the possibility that HDP-mediated changes to the earliest responses after epithelial cell detection of infection could dramatically alter the effectiveness of the early host defence and outcome of infection.

PRRs can function extra- and intracellularly, and include Toll-like Receptors (TLR), NOD-like receptor (NLR), C-type lectin receptors and RIG-I receptors [14, 15]. These receptors can recognise highly conserved pathogen associated molecular patterns (PAMPs) to initiate innate responses to infectious non-self threats, and are critical to effective host defence against infection. In addition to their capacity to modulate TLR signaling, cathelicidins have also been suggested to activate inflammasomes [16, 17]; cytoplasmic multi-protein sensing platforms formed by some NLRs to respond to PAMPs [18]. Inflammasome activation can lead to activation of the cysteine protease caspase 1, processing and release of pro-inflammatory cytokines IL-1β and IL-18, and causing a pro-inflammatory form of cell death, termed “pyroptosis” in macrophages. These responses would be expected to promote an inflammatory response, but their significance in airway epithelial cells is not well understood.

Mechanisms that promote an early and/or enhanced inflammatory response to infection have the potential to stimulate early pathogen clearance and resolution, when appropriately regulated. Our previous studies demonstrated that cathelicidin, whether infection-induced endogenous peptide, or early therapeutically administered peptide, promoted murine pulmonary clearance of *Pseudomonas aeruginosa* [8]. This was the result of cathelicidin-mediated upregulation of early neutrophil recruitment and generation of an enhanced pro-inflammatory response to infection. However, the mechanisms underlying initiation of enhanced inflammomodulatory clearance of this multi-resistant, opportunistic respiratory pathogen remained unknown. We have also demonstrated that cathelicidin modified airway epithelial cell responses to *P*. *aeruginosa* by inducing cell death of infected cells [19], potentially removing a safe niche for these pathogens in the lung. Here we focus on determining the mechanisms that underpin cathelicidin-mediated modulation of airway epithelial cell responses to *P*. *aeruginosa* infection. We show that *P*. *aeruginosa*-infected airway epithelial cells preferentially internalise high levels of LL-37, resulting in lysosomal leakage and cathepsin B-mediated, NLRP3-dependent, activation of caspase-1. These epithelial cells release IL-1β and IL-18 and promote neutrophil influx. This represents a novel mechanism, contrasting significantly with processes in myeloid cells, by which a host defence peptide can provide the second signal required by infection-compromised epithelial cells to promote an inflammasome-mediated protective inflammatory response.

## Results

### LL-37 induces secretion of neutrophil-chemotatic factors during airway epithelial cell infection with *P*. *aeruginosa*

We previously demonstrated that therapeutic airway administration of the human cathelicidin LL-37, in a murine model of pulmonary infection with *P*. *aeruginosa* strain PAO1, promoted pathogen clearance [8]. This enhanced host defence occurred in the absence of any microbicidal effect of LL-37 in the infected lung, by enhancing neutrophil infiltration. Similarly, induction of endogenous murine cathelicidin was essential for an optimal neutrophil response in this pulmonary infection model, and for effective clearance [8]. Importantly, this cathelicidin-mediated protective pro-inflammatory response only occurred in the presence of the bacteria, demonstrating an interaction between infection and HDP-mediated modulation of host defence. However, no cathelicidin-mediated differences in the pulmonary cytokine responses to infection assessed in that study were found. Pulmonary infection with *P*. *aeruginosa* in this murine model leads to extensive exposure of the airway epithelium to this pathogen. *P*. *aeruginosa* can bind and invade (or be internalized by) airway epithelial cells, and induce inflammatory responses [20–23]. Our previous demonstration that LL-37 can modulate the response of airway epithelial cells to *P*. *aeruginosa* infection [19] therefore prompted us to investigate whether these cells could be responsible for enhanced neutrophil recruitment.

**Fig 1:**
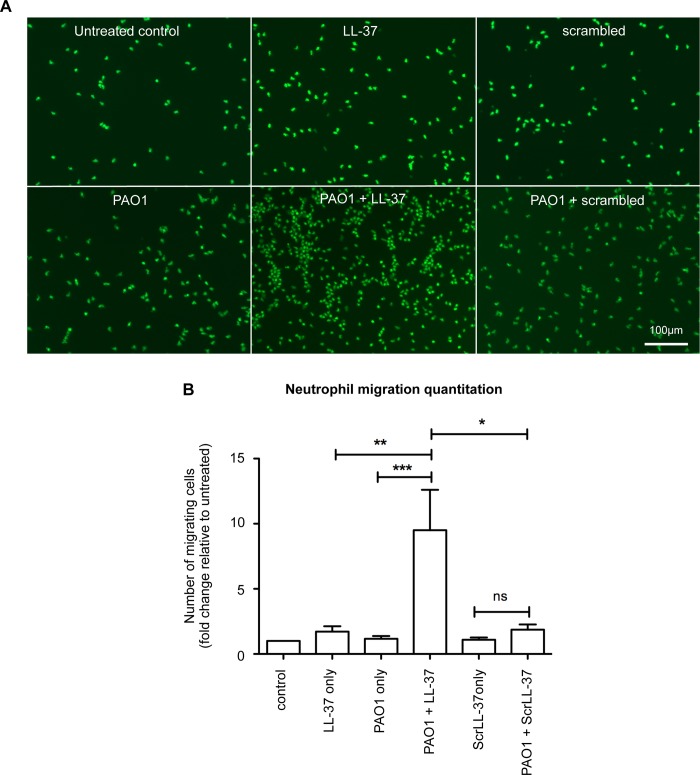
LL-37 induces the release of neutrophil-chemotatic factors from airway epithelial cells during infection with *P*. *aeruginosa*. A) Representative examples of neutrophil migration assayed in ChemoTx chambers. Human neutrophils were assayed migrating towards chambers containing filtered conditioned supernatant from NHBE cells treated for 3 hours with media only (untreated control), 20 μg/ml LL-37, 20 μg/ml ScrLL-37, PAO1 at 10:1 MOI, PAO1 + LL-37 or PAO1 + ScrLL-37. Migrated cells were stained with 1 μM Calcein AM for 15 minutes at 37˚C, and imaged by microscopy. B) Quantitation of migrating neutrophils shown in A, expressed as fold change compared to untreated control. Data represent means +/- SEM from n = 3–6 separate experiments performed with neutrophils isolated from a different donor for each experiment, *** p < 0.001, ** p<0.01, * p < 0.05 versus PAO1 + LL-37 condition, by 2-way ANOVAs with Bonferroni Post-test, to examine the effect of PAO1 infection, with or without LL-37, or scrLL-37 treatment. ns = no significant difference.

An *in vitro* assay was established to analyse the neutrophil chemotactic properties of conditioned media, containing soluble mediators released by primary human bronchial epithelial (NHBE) cells that had been treated for 3 hours with LL-37 and *P*. *aeruginosa* strain PAO1 or controls. This timepoint was selected to examine the early responses of epithelial cells interacting with an invading pathogen, well in advance of any epithelial cell death, and because significant LL-37-enhanced neutrophil influx into the *Pseudomonas aeruginosa*-infected mouse lung had been observed to be well established by 6 hours after infection with LL-37 exposure *in vivo*, implying an early change in signalling responses [8]. Peripheral blood neutrophils, isolated from healthy human donors, were allowed to migrate through a ChemoTx migration chamber towards conditioned media and subsequently quantitated. Significantly more neutrophils migrated towards conditioned media from PAO1-infected NHBE cells exposed to LL-37, than to media from infected cells exposed to a scrambled control LL-37 peptide (Fig 1A and 1B). This was also significantly greater than the responses to media conditioned by NHBE cells exposed to PAO1 or LL-37 alone (which were not significantly above background control level) and greater than the sum of these conditions, demonstrating a synergistic effect compatible with our *in vivo* observations [8]. These data demonstrate that LL-37 can modulate airway epithelial cell responses to *P*. *aeruginosa* infection to induce mediators capable of promoting neutrophil migration to the site of infection.

### LL-37 induces secretion of IL-1β and IL-18 during airway epithelial cell infection with *P*. *aeruginosa*

**Fig 2:**
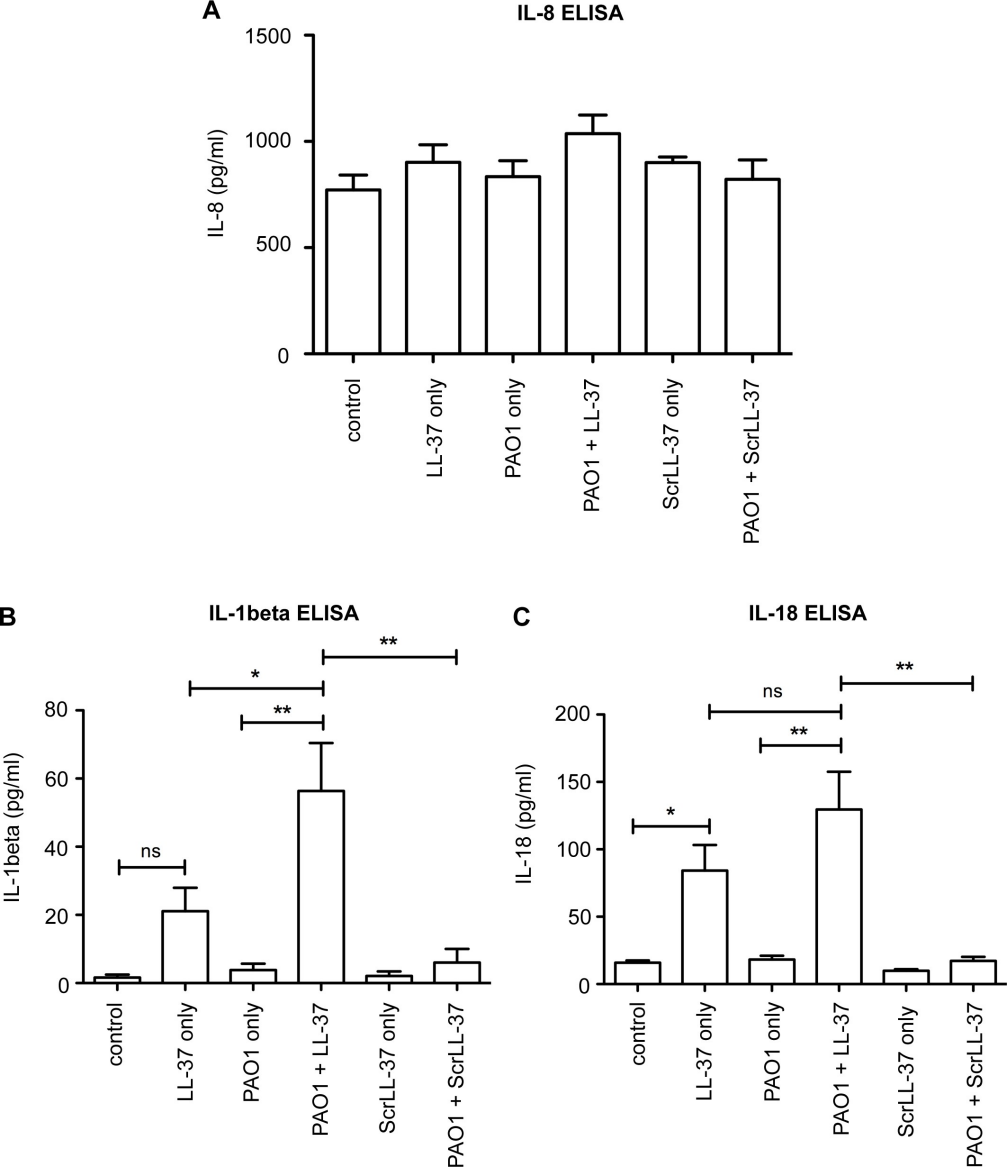
LL-37 induces secretion of IL-1β and IL-18 during airway epithelial cell infection with *P*. *aeruginosa*. ELISA quantitation of human cytokines IL-8 (A), IL-1β (B) and IL-18 (C) released from NHBE cells treated for 3 hours with media only (control), 20 μg/ml LL-37, PAO1 at 10:1 MOI, PAO1 + LL-37, 20 μg/ml ScrLL-37, or PAO1 + ScrLL-37. Data represent means +/- SEM from n = 3 independent experimental repeats, ** p<0.01, * p < 0.05 versus PAO1 + LL-37 condition, by 2-way ANOVAs with Bonferroni Post-test, to examine the effect of PAO1 infection, with or without LL-37, or ScrLL-37 treatment. ns = no significant difference.

To evaluate the differences between conditioned media from PAO1-infected NHBE cells treated with LL-37 and those only infected, or only treated with LL-37 (or exposed to scrambled LL-37), protein microarrays and confirmatory ELISAs were conducted. Whereas no significant difference was seen between the different samples in the levels of IL-8 by ELISA (Fig 2A), or levels of CXCL1/KC, IL-1α, IL-1ra, MIF, or Serpin E1 in protein microarrays (S1 Table), both IL-1β and IL-18 were induced by 3 hours of treatment with LL-37, and secreted at higher levels in LL-37-treated infected cells than in cells treated with LL-37 or PAO1 alone (Fig 2B and 2C). Induction of IL-1β was significantly higher in response to combined stimuli, and greater than the sum of either stimuli individually, with PAO1 alone unable to induce an IL-1β response from these cells. Scrambled LL-37 had no effects, demonstrating the specificity of the LL-37 peptide. These data demonstrate that LL-37 exposure can promote the release of the pro-inflammatory cytokines IL-1β and IL-18 by airway epithelial cells during *P*. *aeruginosa* infection, with the potential to induce cellular inflammatory host responses.

### LL-37 promotes expression of IL-1β by human and murine myeloid cells in a P2X7R-independent manner

**Fig 3:**
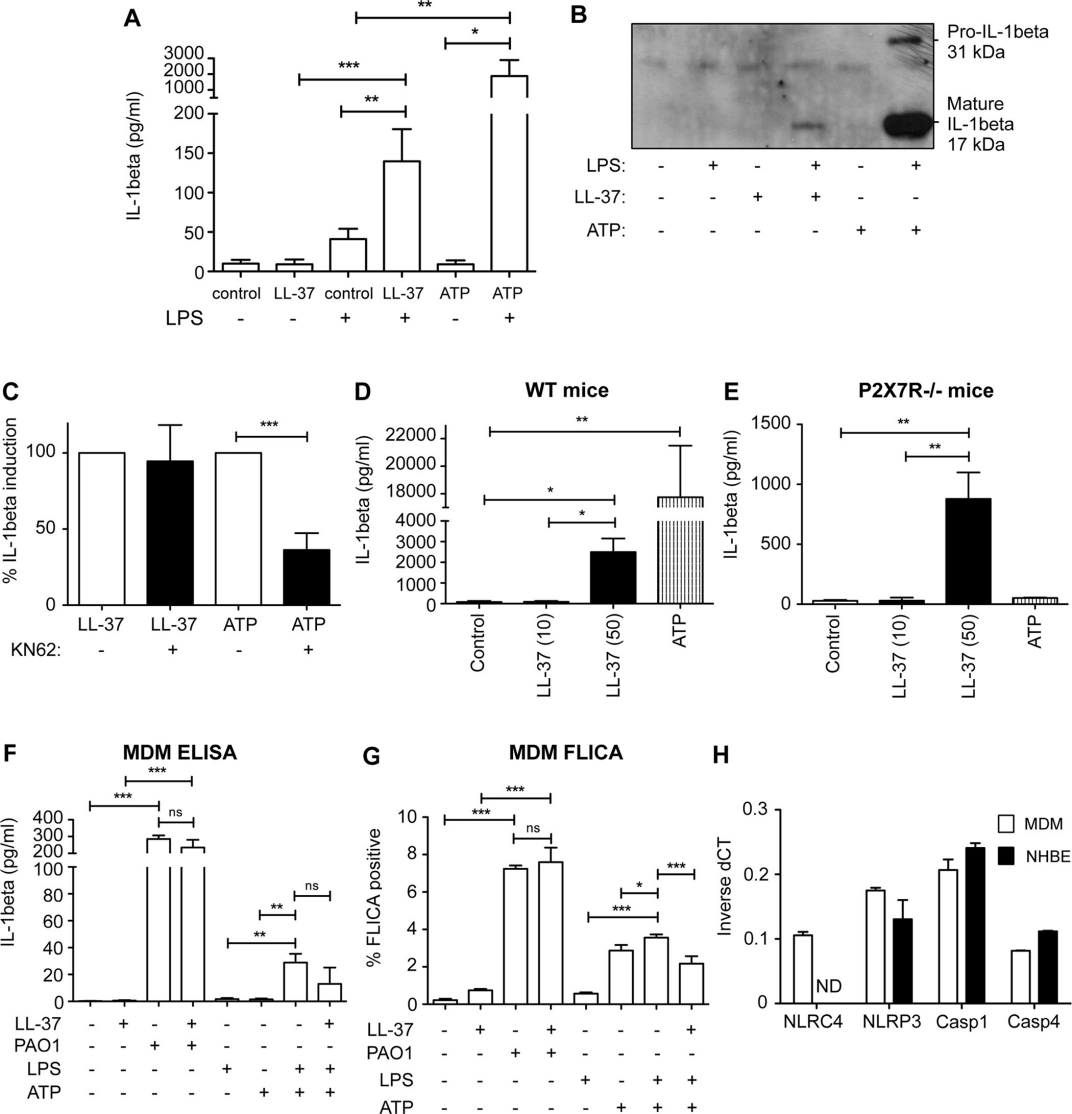
LL-37 promotes expression of IL-1β by human and murine myeloid cells in a P2X7R-independent manner. A and B) Primary human blood-derived monocytes were isolated, pretreated with 10 ng/ml *E*. *coli* 0111:B4 LPS for 3 hours, then exposed for 1 hour to 50 μg/ml LL-37, 5 mM ATP or untreated. Supernatants were assessed for IL-1β by ELISA (A), comparing the effect of LL-37 or ATP on LPS-stimulated or unstimulated cells using 2-Way ANOVAs with Bonferroni post-tests (*** p<0.001, ** p<0.01, * p<0.05, data represent mean +/- SEM for n = 8 per condition), or by western blot (B) detection of pro- and mature IL-1β. (C) Primary human blood-derived monocytes pre-treated with 10 ng/ml LPS as in (A) and also treated with 1 μM KN62 prior to addition of 50 μg/ml LL-37 or 5 mM ATP. Supernatants were assessed for IL-1β by ELISA, and the impact of KN-62 was determined using unpaired t-test (*** p<0.001, data represent mean +/- SEM for n = 4 per condition). D and E) Murine peritoneal macrophages from WT or P2X7R-/- mice were exposed to LL-37 (10 or 50 μg/ml) or ATP (5 mM) for 30 minutes with supernatants analysed by ELISA, and assessed by 1-way ANOVA with Bonferroni multiple comparisons post-test, ** p<0.01, * p<0.05, data represent mean +/- SEM for n ≥ 3 per condition. F and G) Human MDM were treated with either 20 μg/ml LL-37, PAO1 at 10:1 MOI or 10 ng/ml LPS for 3 hours, or 5 mM ATP only for 1 hour, or 10 ng/ml LPS for 3 hours followed by 5 mM ATP for 1 hour +/- 20 μg/ml LL-37. Data represent mean +/- SEM for n = 3–6 per condition, *** p < 0.001, ** p<0.01, * p<0.05, by 2-way ANOVA with Bonferroni Post-test, with supernatants assessed for IL-1β by ELISA (F) and caspase-1 activity assessed by FLICA activity assay (G). ns = no significant difference. H) Real Time PCR detection of inflammasome components using commercial TaqMan Gene Expression assays for NLRC4, NLRP3, caspase 1 (Casp1) and caspase 4 (Casp4) in MDM (white columns) and NHBE (black columns) cells. ND = not detectable.

Previous studies have demonstrated that cathelicidin can promote the release of IL-1β in myeloid cells, in which IL-1β transcription has been induced by pre-treatment with LPS [16, 17]. To confirm these previous observations in our systems, primary human blood-derived monocytes were isolated, pretreated with LPS, and exposed for 3 hours to LL-37 or ATP (as a positive control). As previously described, LL-37 significantly induced the release of IL-1β, although it was a log less effective than ATP (Fig 3A and 3B). However, in contrast to the previous proposal that LL-37 might act directly as an agonist of P2X7 receptor (P2X7R) [16], inhibition of P2X7R had no significant impact on LL-37-mediated induction of IL-1β (Fig 3C). This was in contrast to significant inhibition of ATP-mediated IL-1β release. To confirm this observation, LPS pre-treated peritoneal macrophages from wild type or P2X7R-deficient mice were exposed to LL-37 (Fig 3D and 3E). Exposure to LL-37 induced IL-1β release from both wild type cells and P2X7R-deficient cells, which (as expected) did not respond to ATP. These data confirm the capacity of LL-37 to promote IL-1β by myeloid cells, but indicate a P2X7R-independent mechanism.

Having confirmed the capacity of LL-37 to induce myeloid cell production of IL-1β, we then evaluated the responses to *P*. *aeruginosa* PAO1 and LL-37 in myeloid cells, as a parallel to the conditions applied in the airway epithelial cell experiments. Primary human blood monocyte-derived macrophages (MDM), pre-treated with LPS, expressed IL-1β in response to positive control treatment with ATP, as expected (Fig 3F), although at lower levels than primary monocytes (Fig 3A). Concomitant addition of LL-37 did not further enhance this response. Further treatments were conducted in the absence of LPS pre-treatment, to model the response to live bacteria. Under these conditions, 3 hours after PAO1 infection alone, but not LL-37 alone, exposure had promoted a significant release of IL-1β (Fig 3F). This was not enhanced by the concomitant addition of LL-37. Assessment of caspase-1 activation, as the driver of IL-1β release, was then undertaken using a live cell fluorescent probe (FLICA). Caspase-1 activation in MDM mirrored the release of IL-1β (Fig 3G), with ~35% of LPS pre-treated cells showing activated caspase-1 after 3 hours exposure to ATP, and ~70% of cells activating caspase-1 in response to a 3 hour infection with live PAO1. Responses were not affected by the presence of LL-37 in these cells.

*P*. *aeruginosa* infection is known to activate the inflammasome in macrophages via NLRC4 detection of flagellin and the type-3 secretion system inner-rod protein [24–26]. Involvement of *Pseudomonas* with NLRP3 is less widely reported, but it has been shown to cause activation of NLRP3 to trigger autophagy in macrophages [27], and was shown to activate NLRP3 via mitochondrial perturbation in airway epithelial cells expressing mutant CFTR [28]. More recently, NLRP3 activation was also described in macrophages during longer term (16 hour) infection by *Pseudomonas* via the action of guanylate binding proteins [29]. Thus, to evaluate whether MDM and NHBE cell types both had the required components to facilitate equivalent responses, Q-RTPCR was performed to examine transcription of the genes encoding NLRC4, NLRP3 and caspase-1 and -4 (Fig 3H). Whereas *NLRP3* and *caspase-1* and *-4* were expressed at equivalent levels, no expression of *NLRC4* was detected in NHBE cells, in contrast to MDM. *NLRC4* was also undetectable in the bronchial epithelial cell line 16HBE14o- (S1 Fig). This absence of *NLRC4* expression in bronchial epithelial cells may explain the minimal IL-1β responses of these cells to *P*. *aeruginosa* alone (Fig 2B). Taken together these data suggest that macrophages may be maximally stimulated by infection with *P*. *aeruginosa*, but that airway epithelial cells may need an additional signal to respond. This suggested that the differential *in vivo* host responses to pulmonary infection with *P*. *aeruginosa* in the presence and absence of cathelicidin is more likely related to epithelial cell responses. Therefore, subsequent studies focused on epithelial cells.

### LL-37 induces activation of caspase-1 during airway epithelial cell infection with *P*. *aeruginosa*

**Fig 4:**
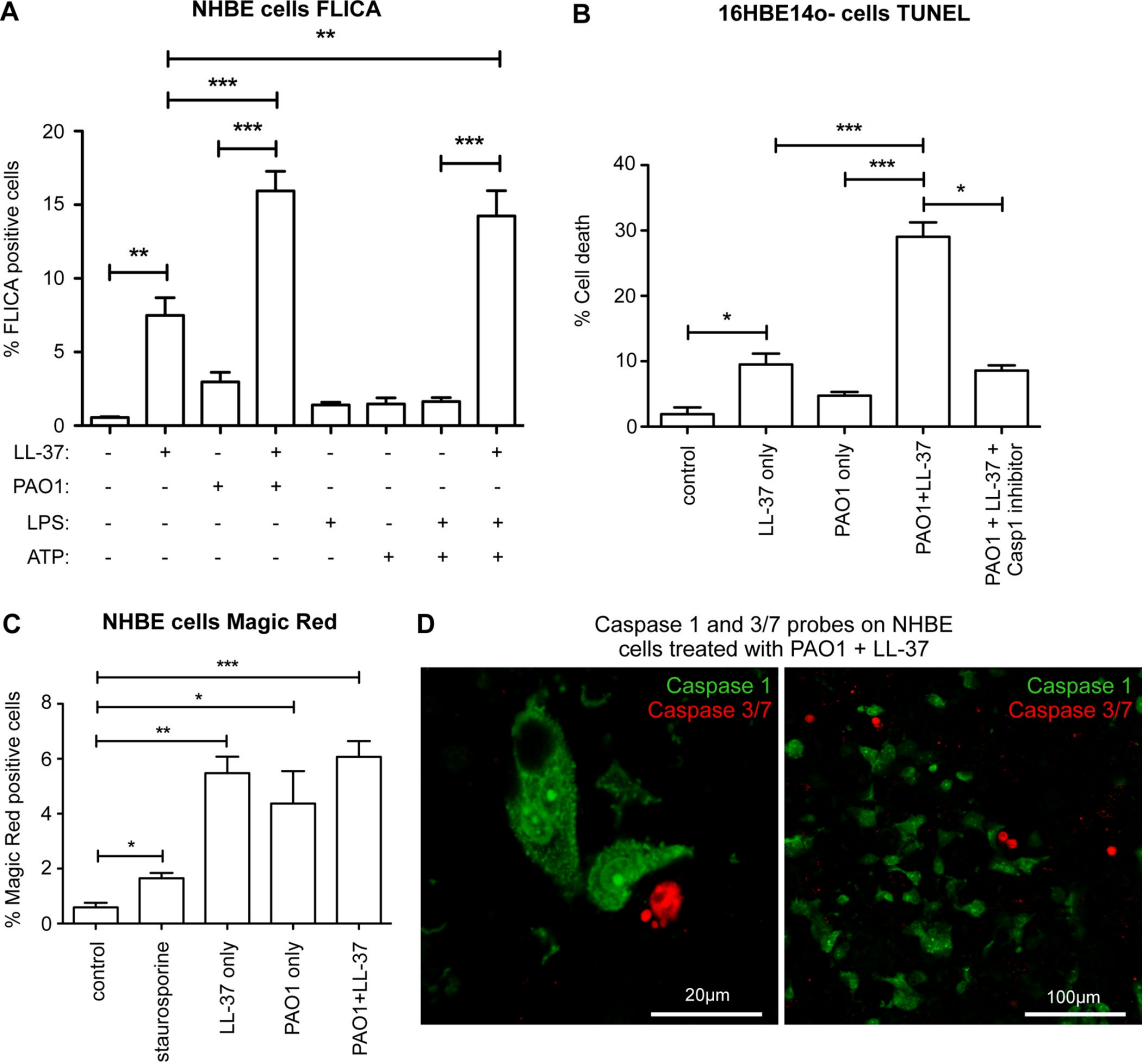
LL-37 induces activation of caspase-1 during airway epithelial cell infection with *P*. *aeruginosa*. A) FLICA Caspase 1 activation assay quantitation in NHBE cells, assessed by fluorescence microscopy, 3 hours post-treatment with either media only (control), 20 μg/ml LL-37, PAO1 at 10:1 MOI, PAO1 + LL-37, 10 ng/ml LPS for 3 hours, 5 mM ATP only for 1 hour, or 10 ng/ml LPS for 3 hours followed by 5 mM ATP for 1 hour +/- 20 μg/ml LL-37. Data represent means +/- SEM from n = 3–6 independent experimental repeats, *** p < 0.001, ** p < 0.01, by 2-way ANOVA with Bonferroni Post-test. B) Quantitation of percentage of cell death at 6 hours post-treatment, assayed by TUNEL staining in 16HBE14o- cells treated with either media only (control), 20 μg/ml LL-37, PAO1 at 10:1 MOI, or PAO1 + LL-37 +/- 25 μM YVAD-CHO Caspase 1 inhibitor. Data represent means +/- SEM from n = 5 independent experimental repeats, *** p < 0.001, ** p < 0.01, * p < 0.05, versus PAO1+LL-37 condition, by 2-Way ANOVA with Bonferroni Post-test, or by unpaired t-test for the separate evaluation of caspase-1 inhibition. C) Quantitation of apoptosis as measured by Caspase 3/7 Magic Red probe in NHBE cells, 3 hours post-treatment with either media only (control), 1 μM staurosporine, 20 μg/ml LL-37, PAO1 at 10:1 MOI, or PAO1 + LL-37. Data represent means +/- SEM from n = 3 independent experimental repeats, *** p < 0.001, ** p<0.01, * p<0.05 by 2-way ANOVA with Bonferroni Post-test, or by unpaired t-test to evaluate the positive control staurosporine. D) Confocal microscopy images, at high and low power magnification, of NHBE cells treated with 20 μg/ml LL-37 and PAO1 at 10:1 MOI, co-stained with Caspase 1 FLICA probe (green) and Caspase 3/7 Magic Red probe (red), showing mutual exclusivity of cell staining with these probes. Representative of n = 4 separate experiments.

The FLICA probe was next used to confirm that the pattern of caspase-1 activation in NHBE cells recapitulated the IL-1β responses. Whereas treatment with LL-37 alone, for 3 hours, caused activation of caspase-1 in a small (~7%) proportion of NHBE cells, *P*. *aeruginosa* PAO1 had a very limited effect (Fig 4A), in agreement with previous studies on lung epithelial responses to *Pseudomonas* [30]. However, in the presence of LL-37, a 3 hour infection with PAO1 led to an increase in caspase-1 activated NHBE cells (to ~16%; Fig 4A) and 16HBE14o- cells (S2 Fig), significantly greater than to LL-37 or PAO1 alone, with the same synergistic pattern observed as for IL-1β release. These responses were a striking contrast to the responses of MDMs (Fig 3G), and suggested the possibility of differential ability to respond to these stimuli. In further contrast to MDM (Fig 3G), LPS pre-treated primary airway epithelial cells did not significantly activate caspase-1 in response to ATP (Fig 4A). However, interestingly, LL-37 also potentiated the responses to ATP in these cells, in manner similar to that observed for *P*. *aeruginosa*. These data suggest that cathelicidin may potentiate inflammasome activation in response to stimuli that are suboptimal activators in airway epithelial cells.

Having previously showed that, over a longer time frame, LL-37 preferentially induced cell death in cells infected with *P*. *aeruginosa* PAO1, in the 16HBE14o- airway epithelial cell line [19], we re-examined this model in light of our new data. Significant LL-37-mediated enhancement of cell death in PAO1-infected cells, assessed by TUNEL staining at 6 hours after infection, also showed a synergistic pattern (Fig 4B). LL-37 treatment with PAO1 infection induced significantly greater cell death (~30%) than in cells treated with LL-37 alone, or those infected without cathelicidin. This could be blocked by the caspase-1 inhibitor, YVAD-CHO, reducing cell death to the level observed in LL-37-only treated cells. Having determined that the synergistic component of this epithelial cell death was capase-1 dependent, a live cell fluorescent probe was used to quantify activation of the apoptosis-inducing caspases-3 and -7, preceding cell death. Low level caspase 3/7 activation was induced in response to stimuli (Fig 4C). However, in contrast to the induction of cell death, the proportion of cells in which these caspases were activated was equivalent in cells irrespective of whether they had received 3 hours exposure to LL-37 or PAO1 alone, or LL-37 exposure concomitant with PAO1 infection (Fig 4C). Using live cell fluorescent probes to evaluate both caspase-1 and caspase-3/7 in the same cells, showed that activation of these caspases was mutually exclusive in cells, with classical condensed morphology seen in apoptotic caspase-3/7 positive cells, and intense caspase-1 foci seen in FLICA positive cells, but no double positive cells seen (Fig 4D). These data demonstrate that cathelicidin-mediated capase-1 activation underpins the LL-37-induced death of *P*. *aeruginosa*-infected cells that we have previously observed [19].

### LL-37 preferentially accumulates in infected epithelial cells, activating caspase-1 via a P2X7R- and FPRL-1-independent mechanism

**Fig 5:**
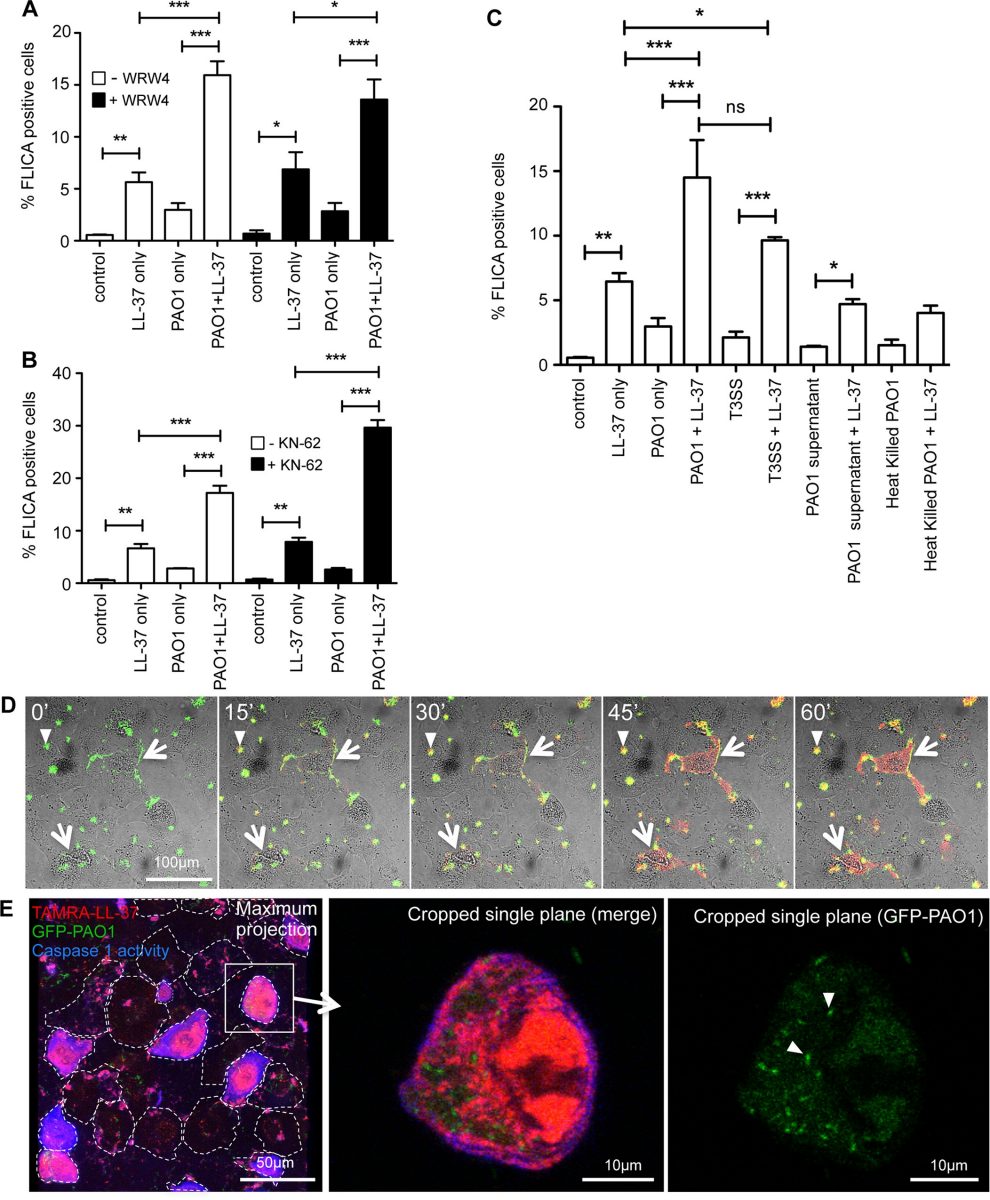
LL-37 preferentially accumulates in infected epithelial cells, activating caspase-1 via a P2X7R- and FPRL-1-independent mechanism. NHBE cells were exposed for 3 hours to media only (control), 20 μg/ml LL-37 only, PAO1/ heat killed PAO1/ *P*. *aeruginosa* strain T3SS at 10:1 MOI +/- 20 μg/ml LL-37, or PAO1 supernatant +/- 20 μg/ml LL-37. Some cells were pre-exposed for 1 hour to FPRL1 inhibitor WRW4 (10 μM) (A), or P2X7R inhibitor KN-62 (20 μM) (B). Caspase 1 activity was measured by FLICA assay (A–C). Data represent means +/- SEM from n = 3 independent experimental repeats, *** p < 0.001, ** p<0.01, * p<0.05, by 2-way ANOVA with Bonferroni Post-test. ns = no significant difference. D) Timelapse series of images (brightfield/fluorescence merge) taken by confocal microscopy showing GFP-PAO1 (green) adhering to NHBE cells (white arrows) with concentration of TAMRA-labelled LL-37 (red), on heavily infected cells. Scale bar = 100 μm. E) Representative maximum projection merged image of a field of NHBE cells stained with GFP-PAO1 (green), TAMRA-labelled LL-37 (red), and Brilliant Violet-YVAD (blue). Scale bar = 50 μm. Inset shows cropped single plane of one NHBE cell demonstrating GFP-PAO1 bacteria (white arrow heads) inside a TAMRA-LL-37 positive cell that is also positive for caspase 1 activation, indicated by Brilliant Violet-YVAD staining. Scale bar = 10 μm.

To determine the mechanism by which LL-37 could activate caspase-1 in airway epithelial cells, previously identified potential receptors for LL-37 were examined. Neither inhibition of FPRL1 (Fig 5A), nor inhibition of P2X7R (Fig 5B), by pre-incubation with WRW4 and KN-62 respectively, were able to inhibit LL-37-mediated activation of caspase-1 during PAO1 infection. The latter data are compatible with our evidence for a P2X7R-independent mechanism of LL-37-mediated IL-1β release in MDM. These findings suggested that modulation of airway epithelial cell caspase-1 activation is mediated by mechanisms other than the classically described LL-37 receptors.

To further characterise the nature of this interaction, the requirement for bacterial secreted factors and/or live bacteria was examined. Substitution of heat-killed PAO1, or bacterial supernatant, for live PAO1, did not replicate the LL-37-enhanced caspase-1 activation (Fig 5C). Although, levels of caspase-1 activation in these cells increased in the presence of LL-37, this was no greater than the response to the LL-37 alone control. However, live PAO1 genetically modified to delete the T3SS operon [31], which renders this *Pseudomonas* strain incapable of invading the cell, retained this function. These data indicate that secreted factors are not sufficient, and that components of the bacterial T3SS are not required, but detection of live *P*. *aeruginosa* by the epithelial cells is sufficient for synergistic activation of capase-1 by LL-37.

*Pseudomonas* can attach to epithelial cell surfaces and can invade (or be internalized by) epithelial cells [21, 23, 32, 33]. Timelapse imaging demonstrated cell-associated aggregates of bacteria rapidly accumulated in infected epithelial cell cultures (Fig 5D; S3 Fig). Subsequent addition of TAMRA-labelled LL-37 to these cultures, 30 min after infection (time “0”), showed the peptide steadily accumulating inside cells associated with large *P*. *aeruginosa* aggregates (white arrows). Using confocal microscopy of live GFP-labelled PAO1, entry of bacteria into the epithelial cells was observed (Fig 5E, white arrow heads) with others accumulating on the surface. Extensive accumulation of TAMRA-LL-37 throughout the cytoplasm (white arrows; Fig 5D) only occurred in cells associated with PAO1 aggregates, and those cells were found to have ~4-fold greater level of associated PAO1 aggregates than cells in which LL-37 had not accumulated (S4 Fig), demonstrating an association between magnitude of infection and high level accumulation of intracellular LL-37. We separately confirmed that the PAO1-infected epithelial cells were not dead prior to addition of LL-37, using the aqueous live cell marker Sytox Green. Sytox Green remained excluded from cells at one hour after infection, at which point TAMRA-LL-37 was added. Subsets of cells were then observed to initially accumulate TAMRA-LL-37 at their membrane, followed by extensive LL-37 uptake into the cytoplasm and Sytox Green positivity (S5 Fig). This may not indicate the onset of cell death, as LL-37 has been shown to enable entry of various materials (including nucleic acid stains and labelled DNA) across the membrane of live cells [16, 34]. However, these experiments demonstrate that the data are not simply LL-37 accumulating non-specifically in dead cells, subsequent to any early bacterially-induced cytotoxicity. To examine the consequence of this, GFP-PAO1-infected, live NHBE cells, exposed to TAMRA-LL-37, were also stained for caspase-1 activation, using a biotin-labelled caspase-1 inhibitor YVAD, followed by brilliant violet-labelled streptavidin (Fig 5E). Confocal imaging showed that caspase-1 activation occurred in the cells with the most bacterial infection and uptake of LL-37. Taken together, these data demonstrate that LL-37 preferentially accumulates inside airway epithelial cells most compromised by infection with live bacteria, and leads to activation of caspase-1 by a P2X7R and FPRL-1 independent mechanism.

### LL-37-mediated activation of caspase-1 during airway epithelial cell infection with *P*. *aeruginosa* is NLRP3-dependent

**Fig 6:**
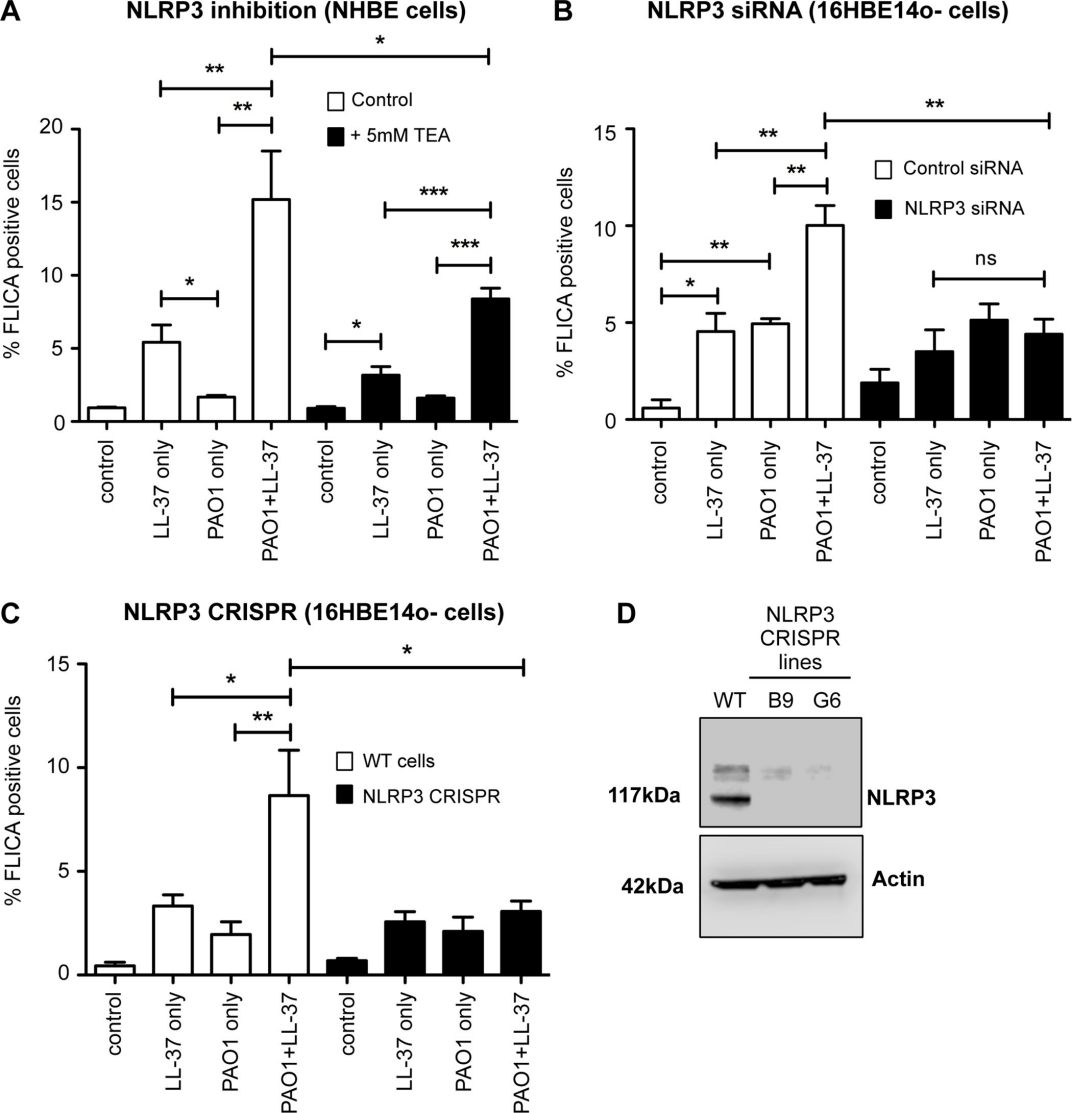
LL-37-mediated activation of caspase-1 during airway epithelial cell infection with *P*. *aeruginosa* is NLRP3-dependent. Airway epithelial cells, either WT NHBE cells (A), NLRP3 siRNA treated 16HBE14o- cells (B), or NLRP3 CRISPR stable 16HBE14o- cell lines (C), were exposed for 3 hours to media only (control), 20 μg/ml LL-37, PAO1 at 10:1 MOI, PAO1 + LL-37. Caspase 1 activity was quantitated by FLICA assay in A) NHBE cells +/- inhibition of NLRP3 by 5 mM TEA, B), 16HBE14o- cells treated with either non-targeting control siRNA (white bars) or NLRP3 siRNA (black bars), C) WT 16HBE14o- cells (white bars) versus NLRP3 CRISPR mutant cells (black bars), following establishment of two CRISPR-targeted NLRP3 mutant cell lines with no detectable NLRP3 expression (demonstrated by western blot of NLRP3 (117 kDa)) in WT cells versus NLRP3 mutant cell lines (D); Actin blot shown as a loading control). In A, B and C, *** p < 0.001, ** p<0.01, * p<0.05, by 2-way ANOVA with Bonferroni Post-test. Data represent mean +/- SEM for n = 3 independent experimental repeats.

To investigate the mechanism by which intracellular LL-37 activated caspase-1 in airway epithelial cells, we examined the importance of NLRP3, previously implicated in LL-37-mediated inflammasome activation in neutrophils [17]. The addition of the NLRP3-inhibiting, potassium ion channel blocker Tetraethylammonium chloride (TEA), to PAO1-infected, LL-37-treated NHBE cultures was found to significantly inhibit peptide-mediated activation of caspase-1 in infected cells (Fig 6A). This was reduced to levels similar to that observed with exposure to LL-37 alone, suggesting a role for NLRP3 in the synergistic induction phenotype. To test the involvement of NLRP3 directly, NLRP3 expression was genetically modified in the 16HBE14o- cell line using both siRNA knock-down (Fig 6B) and CRISPR-mediated knock-out (Fig 6C and 6D) airway epithelial models. NLRP3 siRNA transfection in 16HBE14o- cells resulted in significantly lower levels of caspase-1 activation in PAO1-infected LL-37-treated cells (Fig 6B). To definitively confirm these data, knock-out 16HBE14o- cell lines were generated by CRISPR/Cas9. Two separate mutant lines, B9 and G6 (both confirmed as single base pair insertions causing a non-sense frameshift) were unable to significantly upregulate caspase-1 activation above background stimulation levels upon LL-37 exposure of PAO1-infected cells (Fig 6C). These NLRP3 knock-out cells were shown by western blot to have complete absence of NLRP3 protein (Fig 6D). These data demonstrate that NLRP3 is required for inflammasome-mediated synergistic activation of caspase-1 by uptake of LL-37 into *P*. *aeruginosa* infected airway epithelial cells.

### LL-37-induced activation of caspase-1 in *P*. *aeruginosa* infected epithelial cells, and induction of neutrophil migration, is mediated by lysosomal leakage of cathepsin B

LL-37 is known to interact with cell membranes; permeabilising bacterial outer membranes [35, 36] and the membranes of apoptotic mammalian cells [37, 38]. LL-37 has also been shown to traffic into human airway epithelial cells [39], and specifically to lysosomes and endosomes in THP-1 cells [40]. The release of enzymes cathepsin B and cathepsin D from compromised lysosomal compartments have been shown to induce inflammasome activation [41, 42]. Thus, the extent to which LL-37 could induce lysosomal disruption in infected airway epithelial cells was examined.

**Fig 7:**
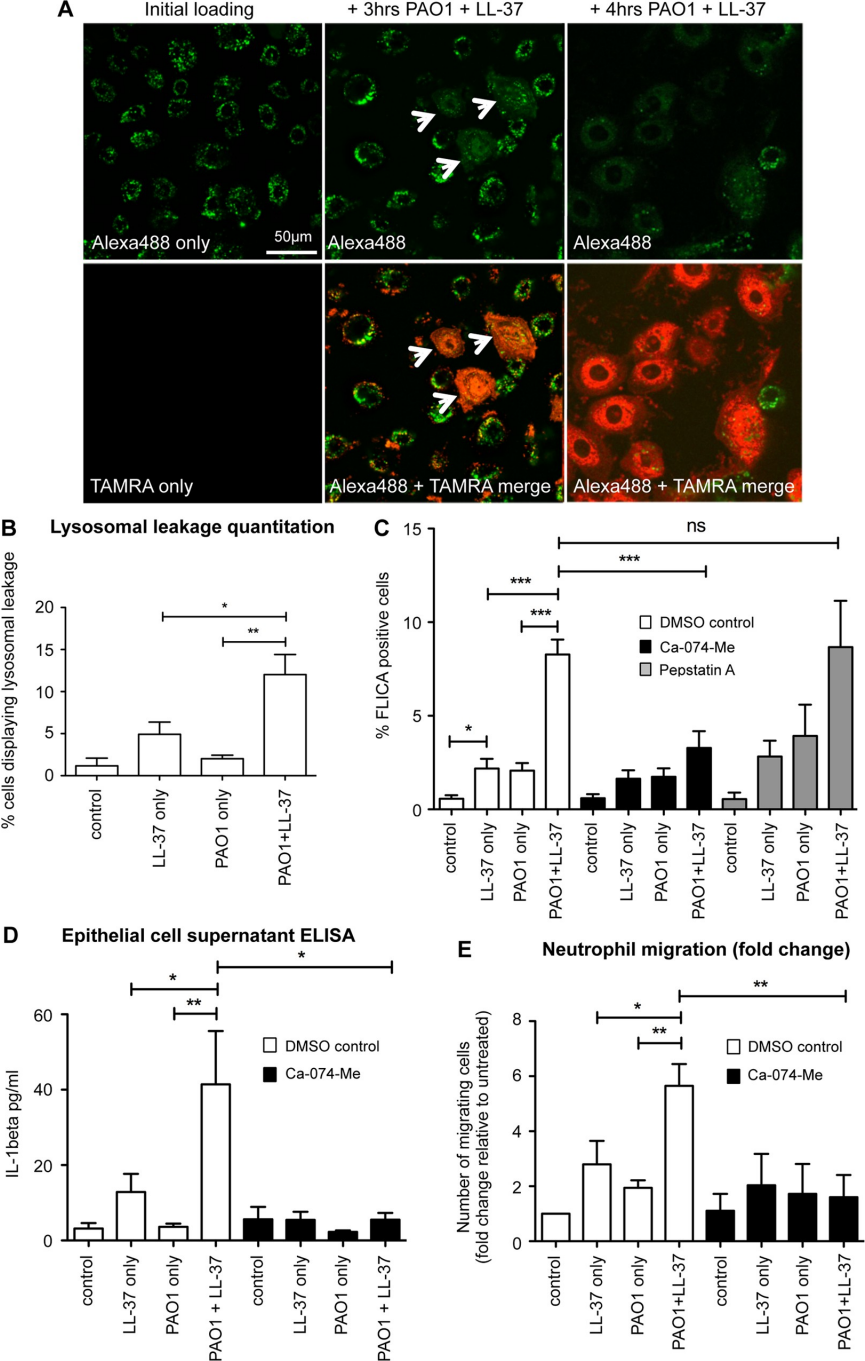
LL-37-induced activation of caspase-1 in *P*. *aeruginosa* infected epithelial cells, and induction of neutrophil migration, is mediated by lysosomal leakage of cathepsin B. A) Confocal microscopy images of NHBE cells loaded with Alexa 488 Dextran (green) +/- treatment with TAMRA-labelled LL-37 (red), with white arrows indicating lysosomal leakage, quantified in (B). ** p<0.01, * p<0.05, data represent mean +/- SEM for n ≥ 3 per condition. C—E) NHBE cells were treated for 3 hours with media only (control), 20 μg/ml LL-37, PAO1 at 10:1 MOI, or PAO1 + LL-37. Some cells were pre-exposed for 1 hour to cathepsin B inhibitor CA-074-Me (20 μM) (C—E), or cathepsin D inhibitor Pepstatin A (20 μM) (C). C) Caspase 1 activation was assessed by FLICA assay D) IL-1β in the supernatant was quantified by ELISA. E) Neutrophil migration in ChemoTx plates towards conditioned supernatant from NHBE cells was assessed. Data represent means +/- SEM from n = 3–6 independent experimental repeats, *** p < 0.001, ** p<0.01, * p < 0.05, by 2-way ANOVA and Bonferroni post-test. ns = no significant difference.

Loading of NHBE cells with fluorescently labelled 10 kDa Dextran resulted in punctate accumulation of fluorescent signal in lysosomes (Fig 7A). Subsequent infection of these cells with PAO1, and exposure to TAMRA-labelled LL-37, resulted in dextran leakage from the lysosomes of a subset of epithelial cells, evidenced by marked diffusion of the fluorescent signal throughout the cell cytoplasm (Fig 7A, white arrows). Visualisation of the TAMRA-labelled LL-37 localisation, demonstrated that the cells with lysosomal leakage were those cells preferentially internalising LL-37 during infection (Fig 7A; red cells). Where cells did not internalise LL-37, the punctate lysosomal location of the dextran remained intact. Quantification of the percentage of epithelial cells with lysosomal leakage (Fig 7B), demonstrated that this was significantly greater in infected cells exposed to LL-37, when compared to LL-37 alone or PAO1 infection alone, recapitulating the pattern for caspase-1 activation.

To evaluate the significance of leakage of lysosomal cathepsin B and D, inhibitors CA-074-Me [43]) and Pepstatin A [44] were used. Whereas inhibition of cathepsin B significantly inhibited the synergistic induction of caspase-1 activation in PAO1-infected primary NHBE cells (Fig 7C) and 16HBE14o- cell (S6 Fig) exposed to LL-37, inhibition of cathepsin D did not. Taken together, these data demonstrate that intracellular LL-37 in PAO1-infected airway epithelial cells, leads to NLRP3 inflammasome-mediated capase-1 activation via induction of lysosomal leakage and the release of cathepsin B.

**Fig 8:**
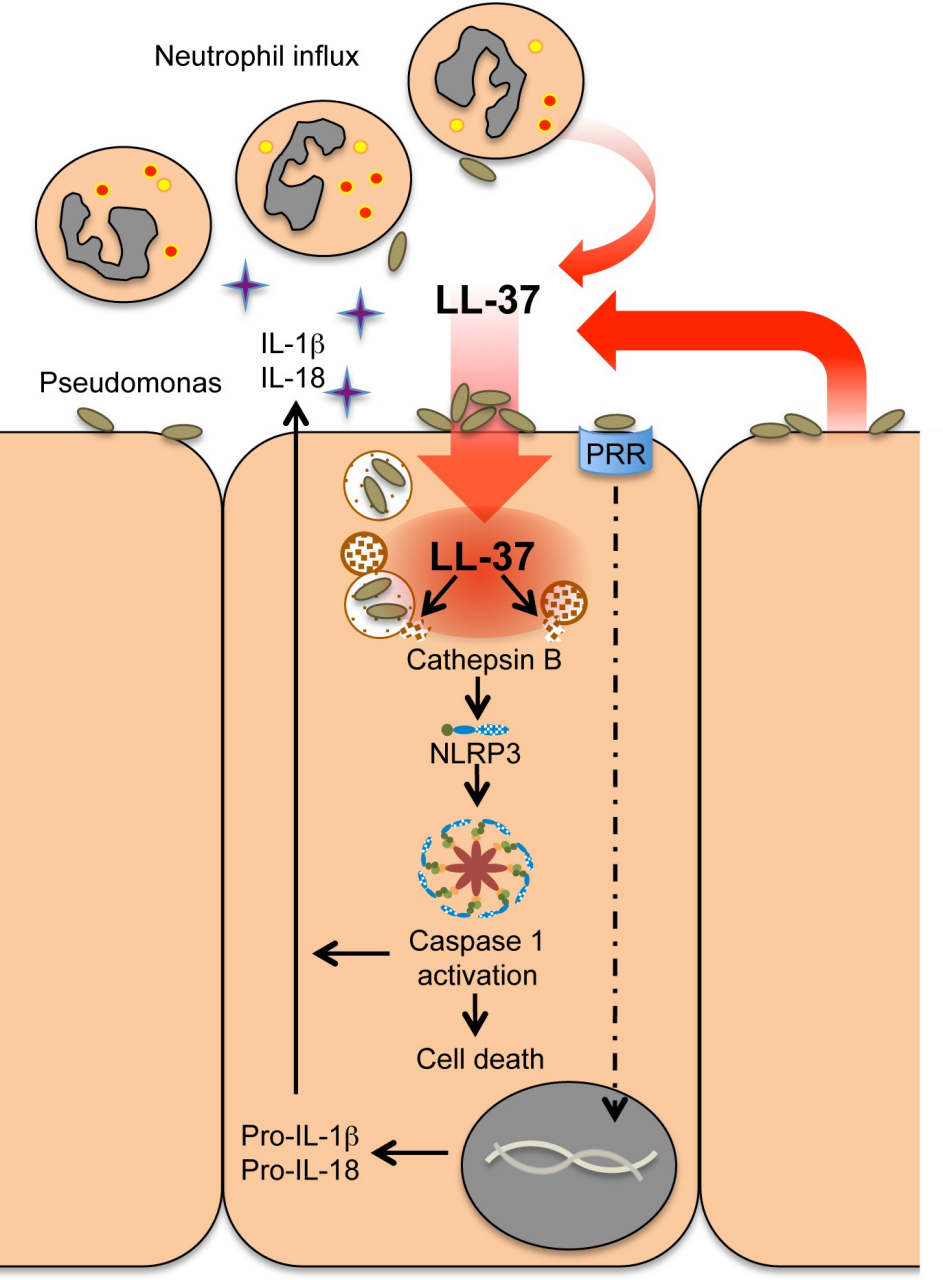
Schematic representation of the mechanism of LL-37-mediated inflammasome activation in Pseudomonas infected airway epithelial cells. Bacterial attachment and internalization into epithelial cells concentrates LL-37 peptide (released by activated cells) into these compromised, infected epithelial cells. Entry of bacteria and cathelicidin into the cell triggers activation of the NLRP3 inflammasome via induction of lysosomal leakage and the release of cathepsin B. Caspase 1 activation in these cells facilitates release of pro-inflammatory cytokines IL-1β and IL-18, promotes recruitment of neutrophils to the affected area, enhances protective inflammation, and may lead to altruistic cell death of the infected cell.

To determine the physiological relevance of lysosomal dependent activation of caspase-1 in these cells, the cathepsin B inhibitor was applied to the initial neutrophil migration assay detailed in Fig 1. NHBE cells were pre-incubated with CA-074-Me cathepsin B inhibitor, before infection with PAO1 and concomitant exposure to LL-37, after which conditioned media was generated and filtered. Inhibition of cathepsin B significantly reduced the level of IL-1β release by LL-37-treated infected cells, to the level of those treated with LL-37 alone (Fig 7D). Application of these conditioned media to the primary human neutrophil migration assay, demonstrated that cathepsin B inhibition blocked LL-37-promoted production of neutrophil chemotactic factors by PAO1-infected airway epithelial cells. (Fig 7E). These data demonstrate that the enhanced neutrophil chemotactic capacity of airway epithelial cell conditioned media, results from LL-37-mediated activation of the NLRP3 inflammasome and caspase-1 in *P*. *aeruginosa*-infected cells, operating via a mechanism of lysosomal leakage of cathepsin B (Fig 8).

## Discussion

*Pseudomonas aeruginosa* is an important, opportunistic, multidrug-resistant human pathogen. It is associated with a wide range of serious acute and chronic infections, including ventilator-associated pneumonia and sepsis syndromes, and is the predominant pulmonary infection leading to fatal deterioration of lung function in patients with CF [45]. New interventional approaches for the treatment of *P*. *aeruginosa* are urgently needed in the context of the increasing global threat of antimicrobial resistance, and may be informed by a greater knowledge of effective innate host defence. Cathelicidins are a vital, non-redundant antimicrobial host defence peptide component of innate host defence, necessary for *in vivo* protection against infections of the lung, skin, intestinal tract, urinary tract and eye (reviewed in [7]). We have previously demonstrated that innate antimicrobial host defence peptides of the cathelicidin family can enhance the clearance of pulmonary *P*. *aeruginosa in vivo* by amplifying the protective neutrophilic inflammatory response [8]. However, the mechanism by which cathelicidin promotes protective pulmonary inflammation in the context of an infectious threat, but not when delivered to the quiescent lung, remained unclear.

Here we describe a novel mechanism to promote innate host defence against *P*. *aeruginosa* infection, mediated by a modulatory function of the cathelicidin LL-37 upon infected airway epithelial cells. Unless rapidly cleared by professional phagocytes, *P*. *aeruginosa* entering the lung will predominantly encounter airway epithelial cells, to which this pathogen can adhere and invade, or be internalised; a process that could be a pathogenic mechanism to avoid host-mediated killing, or a host defence mechanism [21, 23, 32, 33, 46–48]. Under permissive conditions, this can create a safe niche for survival and intracellular proliferation of the bacteria in compromised epithelial cells [47]. However, the release of danger signals, coupled with altruistic cell death to remove a small number of infected cells which have internalised multiple organisms, could protect the host [19, 48, 49]. In contrast to professional phagocytes, a relatively quiescent epithelium is desirable, unless the initial host defence has been overwhelmed and the epithelial barrier is compromised by infection. In this context, a requirement for multiple signals to licence significant epithelial cell induction of a robust neutrophilic response in the lung may be a necessary protective safeguard.

We demonstrate that *P*. *aeruginosa* infection of airway epithelial cells *in vitro* does not activate the inflammasome without an additional signal, perhaps due to an absence of NLRC4 expression. However, infection in the presence of cathelicidin promotes caspase-1 activation, release of IL-1β and IL-18, and the influx of neutrophils. Whether the key source of this cathelicidin is activated local macrophages or epithelial cells (with potential responses to cathelicidin produced by the infected epithelial cell itself, or by its neighbours), or degranulating neutrophils entering the site in an escalating inflammatory response in the lung, remains to be determined. An additional limitation of this study was the use of submerged airway epithelial cell cultures. While both undifferentiated primary cells and an immortalised cell line showed the same responses to stimuli, we cannot exclude the possibility that cells differentiated at air-liquid interface could respond differently. Regardless, the synergistic activity of infection and peptide exposure in these cells was found to be mechanistically dependent upon interaction of the epithelial cells with live *P*. *aeruginosa*, but not dependent upon T3SS, and to be dependent upon cathelicidin-mediated release of cathepsin B from lysosomes or phagolysosome in infected cells, inducing activation of the NLPR3 inflammasome. The process by which infection increased the uptake of LL-37 into those compromised epithelial cells, remains the focus of ongoing work. In this regard, the significance of Pseudomonas-induced ceramide-enriched lipid platforms, will be of interest; having critical roles in pathogen internalisation, NF-κB activation and IL-1β transcription in response to the infection [46, 50]. Once inside the cell, LL-37 has been shown to traffic to lysosomes and endosomes in THP-1 and epithelial cells [39, 40], is known to localise in and cross cellular membranes, and can induce pore formation in apoptotic cell membranes [37, 38]. Release of cathepsin B and cathepsin D from compromised lysosomal compartments has been shown to induce the activation of inflammasomes [41]. In addition, the cathepsin B inhibitor CA-074-Me has been shown to inhibit activation of the NLRP3 inflammasome [43]. In our studies, the effects of LL-37 were blocked by a cathepsin B inhibitor, with no impact of cathepsin D inhibition. Although inflammasome activation was required for the synergistic danger response observed in infected, peptide-treated cells, parallel effects of cathelicidin may also contribute to this process. In that regard, it is interesting to note that LL-37 also potentiated airway epithelial cell activation of caspase-1 in response to ATP, to which, in contrast to myeloid cells, airway epithelial cells were largely unresponsive. Given this apparent capacity to potentiate suboptimal inflammasome-activating signals in airway epithelial cells, the impact upon infection with other, clinical isolates of *P*. *aeruginosa*, and potentially also infections with other bacteria and/or viruses, will be of great future interest. Regardless, this synergistic activation of inflammasome-mediated neutrophilic inflammation is compatible with our *in vivo* observations; in which endogenous cathelicidin was required for maximal neutrophil-mediated inflammation, and therapeutic delivery of LL-37 enhanced neutrophil responses to *P*. *aeruginosa* infection [8]. It is also worth noting that while LL-37 alone was capable of inducing low level NLRP3-independent caspase-1 activation in our epithelial cell studies (Fig 6), this was not sufficient to promote significant neutrophil chemotaxis (Fig 1). Similarly, delivery of LL-37 alone (in the absence of infection) to the murine lung did not induce pulmonary neutrophil influx *in vivo* [8]. Our new *in vitro* data now justify future mechanistic *in vivo* research to examine the impact of cathelicidin on release of IL-1β and IL-18 responses in murine models of pulmonary *P*. *aeruginosa* infection, to determine the factors modified by induction of these cytokines to act as the direct chemokines for neutrophils in our *in vitro* and *in vivo* models, and the effect of NLRP3 deficiency on the efficacy of therapeutic administration of cathelicidin.

Inflammasomes have been widely investigated in immune effector cells, particularly macrophages, but are less well studied in epithelial cells. Nevertheless, recent research has shown the biological importance of epithelial cell inflammasome-mediated responses against pathogens (summarised in [51]). Of particular significance, studies in the gut have shown that inflammasome activation and pyroptosis are important in host defence against Salmonella infection, helping to clear pathogens by removing an intra-cellular replication niche [52]. Although LL-37-mediated cell death in our *P*. *aeruginosa* infected airway epithelial cells was caspase-1 dependent, the role of non-canonical pathways and gasdermin-D in this system remain to be determined. It is therefore unclear whether this cell death should be described as a form of epithelial cell pyroptosis. Furthermore, the relative significance of this, versus upregulated neutrophil responses, to the cathelicidin-enhanced protection against pulmonary *P*. *aeruginosa in vivo* remains to dissected in future studies of lung infection models.

Evidence suggests that inflammasome activation can have significant consequences of pulmonary diseases [53, 54], but the relative impacts of acute, potentially essential, protective activation, versus inappropriate, potentially damaging, chronic activation remains to be fully elucidated. Despite this, it is clear that early innate immune responses of airway epithelial cells constitute an important component of first line of defense against respiratory disease, and, compatible with our data, lung epithelial cells can secrete IL-1β and IL-18 to induce the inflammatory response [51]. Although not of the same magnitude as myeloid cells in our studies, the combined effect of epithelial cell cytokine production at sites of infection may be pivotal. Furthermore, in addition to its capacity to directly stimulate the production of chemokines by peripheral blood monocytes and lung epithelial cells [55, 56], LL-37 can also synergistically enhance IL-1β-mediated production of cytokines and chemokines [57]. Similarly to the properties described in our manuscript, this potentiation was observed using concentrations of cathelicidin observed in the inflamed the human lung *in vivo* [58], suggesting a mechanism for further enhancement of the impact of the cathelicidin-mediated induction of IL-1β described herein, in the context of pulmonary inflammation.

In conclusion, we therefore propose that cathelicidin-mediated NLRP3 inflammasome activation in infected airway epithelial cells represents activation of a “fire-alarm”; triggered by escalating pulmonary inflammation to an overwhelming threat that necessitates epithelial-generated enhanced neutrophilic inflammation and potentially the altruistic death and removal of compromised infected epithelial cells. This represents a novel modulatory function of an important innate antimicrobial host defence peptide, with the potential to open opportunities for the development of HDP-based therapeutics (or inducers) that can combine microbicidal activity with immunomodulatory / “innate immune adjuvant” function. Such approaches aim to harness the most effective, evolutionary-tested successes of the innate immune system as alternative or complementary approaches to our conventional therapies, avoiding issues of rapid generation of resistance by promoting pathogen clearance indirectly by modulatory properties of the HDP. This “immunomodulatory antimicrobials” approach has the potential to revolutionise our strategies to infectious diseases in a manner that could parallel the recent successes seen in cancer immunotherapy, and is of high significance in the context of the threat of antibiotic-resistance.

## Methods

### Ethics statement

For leukocytes isolated from healthy volunteer blood, informed written consent was obtained from all subjects, AMREC reference no. 15-HV-013. All animal experiments were carried out in accordance with the United Kingdom Animals (Scientific Procedures) Act 1986 and approved by the Home Office and the local Animal Ethical Review Group, University of Manchester, project license number 403076.

### Peptides, antibodies and inhibitors

LL-37 (LLGDFFRKSKEKIGKEFKRIVQRIKDFLRNLVPRTES; MW 4493.33) and scrambled LL-37 control peptide (ScrLL-37) (RSLEGTDRFPFVRLKNSRKLEFKDIKGIKREQFVKIL) were custom synthesised by Almac (East Lothian, Scotland) using Fmoc solid phase synthesis and reversed phase HPLC purification. Peptide identity was confirmed by electrospray mass spectrometry, purity (>95% area) by RP-HPLC and net peptide content determined by amino acid analysis. Lyophilised peptides were reconstituted in endotoxin free water at 5 mg/ml stock concentration, determined to be endotoxin-free using a Limulus Amebocyte Lysate Chromogenic Endotoxin Quantitation Kit (Thermo Scientific, UK), and stored at -20 °C. Peptide functionality was confirmed by assessing anti-endotoxic activity.

IL-1beta antibody was from R&D systems and used at 100 ng/ml, anti-NLRP3/NALP3 antibody was used at 1:500 dilution, from Caltag Medsystems (Buckingham, U.K.; Cat. No. AG-20B-0014-C100); anti-Beta-Actin antibody was used at 1:2000, from Sigma Aldrich (Merck group, Darmstadt, Germany; Cat. No. A1978).

Caspase 1 inhibitor YVAD-CHO and the Formyl Peptide Receptor-Like 1 antagonist WRW4 were from Calbiochem (Merck group, Darmstadt, Germany). Cathepsin B inhibitor CA-074-Me was from Enzo Life Sciences (Exeter, U.K.). Cathepsin D inhibitor Pepstatin A, Tetraethylammonium chloride (TEA), the purinergic receptor P2X7R inhibitor KN62, and Staurosporine were from Sigma Aldrich.

### Cell and bacterial culture

Normal Human Bronchial Epithelial (NHBE) primary cells (Lonza, Basel, Switzerland) from two donors were purchased, cultured and maintained in BEBM media with supplements (Lonza) and used throughout. No significant donor-dependent variation was observed. 16HBE14o^-^ cell line (transformed human bronchial epithelial; a kind gift from Dieter Gruenert at the University of California San Francisco) was maintained in DMEM containing 10% FBS, 2 mM L-glutamine and 1% Penicillin/Streptomycin (Pen/Strep). Cells were incubated in a 37°C incubator with humidified atmosphere of 5% CO_2_, and grown on flasks or chamber slides previously coated with a solution of Collagen (Cultrex, Trevigen, Gaithersburg, U.S.A.; 0.05 mg/ml) and Fibronectin (Sigma-Aldrich; 0.1 mg/ml) syringe-filtered through a 0.22 μm filter.

Transfection of epithelial cells was performed using Lipofectamine 2000 (Invitrogen, Carlsbad, U.S.A.) by following the manufacturers’ instructions. For transient transfection, cells were transfected 48–72 hours prior to use. Transfection of control siRNA and NLRP3 siRNA cells were performed using Lipofectamine 2000 reagent by following the manufacturers’ instructions. Cells were transfected 48–72 hours prior to use.

*P*. *aeruginosa* strain PAO1 was a gift from J. R. W. Govan (University of Edinburgh). GFP-PAO1 was a gift from T. Tolker Nielsen (University of Copenhagen), and PAO1-dT3SS was a gift from E. Gulbins (University Duisburg-Essen, Germany).

### Isolation of human leukocytes

Peripheral blood neutrophils and monocytes were isolated from healthy human volunteers via dextran sedimentation and Percoll discontinuous gradients as described [59]. Neutrophils were used immediately in ChemoTx (Neuro Probe Inc., Gaithersburg, U.S.A.) migration assay plates, at a concentration of 5 x 10^4^ in PBS on the upper side of the membrane as per manufacturer’s instructions, and allowed to migrate towards conditioned BEBM media from NHBE cells. Conditioned media was made by treating NHBE cells for 3 hours with LL-37, scrambled LL-37 or *Pseudomonas aeruginosa* PAO1, either alone or in combination (all as detailed in Fig 1), removing cell supernatant and filtering through a 0.22 μm-filter. Cells were allowed to migrate for 1 hour at 37˚C, and migrated cells in the lower well were then stained with 1 μM Calcein AM for 15 minutes before visualization/quantitation on an ‘EVOS fl’ fluorescence inverted microscope (Fisher Scientific, Loughborough, U.K.).

Peripheral blood mononuclear cells were incubated at 4 x 10^6^/mL in IMDM (PAA Laboratories, Somerset, UK) at 37°C, 5% CO2, for 1 hour. Non-adherent cells were removed and adherent monocytes were either used immediately, or cultured for 6 days in IMDM with 10% autologous serum to generate monocyte-derived macrophages before treatment. Cells were either primed with LPS (*E*. *coli* 0111:B4 Ultrapure, Invivogen (Toulouse, France); 10 ng/ml) for 3 hours prior to addition of 5mM ATP (Sigma Aldrich) as required, or treated with LL-37 or ScrLL-37 (20–50 μg/ml) or PAO1 at a Multiplicity of Infection (MOI) of 10:1 as described in the text. Cells were then used for preparation of RNA by RNeasy kit (Qiagen, Manchester, U.K.), and cell supernatant used for cytokine analysis by ELISA as described below.

### Murine peritoneal macrophages

Macrophages were prepared from adult male C57BL/6 (WT) mice (Harlan) and P2X7R KO mice [60] as described previously [61]. In brief, the peritoneal cavity was lavaged with RPMI 1640 media (Sigma). Cells were collected by centrifugation (250 x g, 5 minutes) and plated in 24-well plates at a density of 5 x 10^5^ cells/well in RPMI 1640 media (Sigma) supplemented with 5% FBS (PAA Laboratories), 100 units/ml penicillin, and 100 μg/ml streptomycin (Sigma). Cells were cultured overnight (37 °C, 5% CO_2_) before non-attached cells were removed by a media change. Cells were incubated with LPS (1 μg/ml for 2 hours) before treatment with LL-37 and ATP as indicated.

### Measurement of Caspase 1, Caspase 3/7 activity and TUNEL staining

16HBE14o- or NHBE cells were grown overnight at a seeding concentration of 0.8 x 10^5^ per well of a collagen/fibronectin-coated chamber slide. Prior to treatments below, cells were washed twice in PBS (Gibco) and media was replaced with 250 μl serum-free BEBM (Lonza; for NHBE cells) or DMEM (Gibco, Thermo Fisher UK; for 16HBEo- cells) and returned to 37˚C. PAO1 bacteria were grown in Luria Bertani (LB) Broth overnight at 37˚C with shaking to achieve a stationary-phase suspension. Before use, bacterial suspensions were diluted 1:5 in fresh LB broth and were returned to incubate at 37˚C for a further 2 hours to reach log phase. Bacterial cultures were centrifuged at 1885 x g for 10 minutes, and bacterial supernatant was removed and filter-sterilised through a 0.22 μM filter for use as required in the text. PAO1 were re-suspended in PBS, and diluted to an optical density (OD) 600 of 0.1, equating to 10^8^ bacteria/ml. If required, bacteria were then heat killed by incubation at 70˚C for 30 minutes. PAO1 were then added to cells in serum-free media at 10:1 MOI. LL-37 or control scrambled peptide was added at the concentrations indicated in the text. If required, cells were pre-incubated for 1 hour at 37˚C with inhibitors as indicated in the text, prior to addition of PAO1/peptide. Cells and PAO1 were then returned to 37˚C for 3 hours.

Caspase 1 and Caspase 3/7 activity in live cells was assessed using FLICA and Magic Red fluorescent probes respectively (ImmunoChemistry Technologies, Bio-Rad AbD Serotec, Kidlington, U.K.), as per manufacturer’s instructions, in an incubator at 37˚C for 1 hour, with the addition of 1 μg/ml Hoechst (Life Technologies) for the final 30 minutes. Cells were then viewed using an EVOS fl inverted fluorescence microscope (Thermo Fisher U.K.) and analysed using ImageJ software. Hoechst staining was used to calculate total cell number per field, and Caspase 1 or 3/7 positivity of cells was manually assessed and counted for each field. TUNEL staining was performed with *In Situ* Cell Death Detection Kit (Roche Applied Science, West Sussex, UK), according to manufacturer’s instructions, 6 hours after treatments.

### Lysosomal leakage

NHBE cells seeded onto chamber slides were loaded for 3 hours with 20 μM Alexa 488 Dextran, 10kDa (Life Technologies) at 37˚C in a humidified incubator, prior to treatment with media only, 20 μg/ml LL-37 (or TAMRA-labeled LL-37), PAO1 at 10:1 MOI, or PAO1 + LL-37 for a further 3 hours. Cells were then imaged live using a Leica SP5 confocal microscope. Acquired images were subsequently analysed for evidence of lysomal leakage, indicated by a diffusion of the green fluorescent signal throughout the cell cytoplasm, compared to bright punctate fluorescence in media-only control conditions. Quantitation of leakage was performed by expressing the number of cells displaying diffused green fluorescence as a percentage of the total number of cells in that field, across a minimum of 5 fields per experiment.

### Western blotting

Cells were lysed with MPER lysis buffer (Life Technologies), centrifuged at 17,740 x g at 4 ˚C for 10 minutes, and protein concentration determined by BCA assay. Equalized samples were loaded onto 4–12% pre-cast polyacrylamide gel (Novex NuPage, Life Technologies), transferred to a Nitrocellulose membrane (Life Technologies), blocked for an hour with 5% skimmed milk dissolved in TBS + 0.05% Tween-20 (TBS-Tw), and then incubated with primary antibodies as indicated in the text in blocking solution overnight at 4˚C. After washing in TBS-Tw, membranes were incubated with appropriate secondary HRP-labelled antibodies (DAKO, Agilent, Santa Clara, U.S.A.) in blocking solution, treated with ECL Prime substrate (Sigma-Aldrich) in accordance with manufacturer’s instructions, and imaged on CL-Xposure film (Thermo-Scientific).

### ELISA

Cells (MDM, NHBE or 16HBEo-) were plated at 1.5 x 10^6^ cells in 1 ml complete media in a 6-well plate and were then incubated overnight at 37˚C. Cells were infected for 3 hours with PAO1 and LL-37, as per Caspase 1 activity section above. PAO1 was used at 10:1 MOI, LL-37 peptide was used at 20 μg/ml. Supernatants were collected and stored at -20 ˚C until use. The concentration of IL-1β, IL-18 and IL-8 in cell supernatants was measured by ELISA kit (eBioscience, Thermo Fisher U.K.), according to the manufacturer’s instructions.

### qRT-PCR

RNA was made from treated cells using RNeasy mini kit (Qiagen), according to manufacturer’s instructions, and DNase treated with RQ1 DNase (Promega, Wisonsin, U.S.A.) for 30 min at 37˚C. cDNA was prepared using TaqMan reverse transcriptase kit (Life Technologies). Quantitative Real Time PCR was performed on a StepOne Real Time PCR machine (Life Technologies), using Gene Expression Mastermix and TaqMan gene expression assays for Caspase 1 (Assay ID Hs00354836_m1), Caspase 4 (Assay ID Hs01031951_m1), NLRP3 (Assay ID Hs00918082_m1), NLRC4 (Assay ID Hs00892666_m1) and 18S (Assay ID 4319413E-1403063) (all Life Technologies), as indicated in the text.

### Construction of knock-out 16HBE14^o-^ by CRISPR/Cas9

Two target sites for NLRP3 gRNA were designed according to the Zhang laboratory online CRISPR designing tool [62]. Target sequences were cloned into vector pSpCas9(BB)-2A-GFP (PX458), a gift from Feng Zhang (Addgene plasmid # 48138; http://n2t.net/addgene:48138; RRID:Addgene_48138), and transfected into 16HBE14o- cells with Lipofectamine 2000 (Invitrogen). Cells were flow sorted using a BD Biosciences FACS Diva 8.0.1 into single cells per well of a 96-well plate, gating for live single cells and GFP-positivity. Isolated GFP-positive cells were expanded, and tested for mutation by PCR and sequencing across the target area. Two clones, B9 and G6, were identified as having single base pair insertions at the target site causing a non-sense mutation that abolished NLRP3 protein production, as demonstrated by Western blotting.

### Confocal microscopy

For microscopy, other than in Caspase 1 FLICA and neutrophil migration experiments described above, images were acquired on a Leica SP5 confocal microscope, with 63x oil objective. Cells were grown on Nunc LabTekII chamber coverslides (Sigma Aldrich), pre-coated with Collagen and Fibronectin, overnight at 37˚C. Cells were imaged live after incubation with GFP-PAO1, TAMRA-LL-37 (Almac), Biotin-YVAD-CMK (Cambridge Bioscience, Cambridge, U.K.) and Brilliant Violet 421 Streptavidin (BioLegend, San Diego, U.S.A.), FLICA Caspase 1 probe or MagicRed Caspase 3/7 probe (both ImmunoChemistry Technologies) or Alexa 488 10kDa Dextran (Cat. No. D-22910, Life Technologies), as indicated in the text. Images were acquired using Leica Application Suite software, and subsequently processed (cropping/brightness-contrast only) in Adobe Photoshop CS5.1 software.

### Statistics

Statistical analysis was performed using the GraphPad PRISM statistical package (GraphPad software, La Jolla, USA) by 2-way ANOVA with Bonferroni Multiple Comparison Post-test (Figs 1B, 2A–2C, 3A, 3F, 3G, 4A–4C, 5A–5C, 6A–6C and 7B–7E, and Supporting S3 and S6 Figs), 1-way ANOVA with Bonferroni Post-test (Fig 3D and 3E), or unpaired t-test (Figs 3C and 4B between last 2 columns, 4C between first 2 columns), as stated in the respective figure legends. p-values below 0.05 were considered significant. Figures show mean +/- SEM.

## Supporting information

S1 Table R+D Cytokine Profiler blot quantitation. Quantitation of additional cytokines released from NHBE cells after treatment. A Cytokine Profiler dot blot (R+D Systems) was probed with filtered supernatant from NHBE cells treated for 3 hours with either 20 μg/ml LL-37 or 20 μg/ml LL-37 + PAO1 at 10:1 MOI. Quantitation was performed by measuring Integrated Pixel Density of each cytokine spot in ImageJ. S1 Table lists the mean Integrated Pixel Density values for 4 measurements from each cytokine in each condition.
(TIF)

S1 Fig Real Time PCR (16HBEo- cells). Quantitative Real Time PCR of inflammasome components NLRC4 and NLRCP3 in 16HBE14o- cells, showing no detectable NLRC4. Data represent means +/- SEM from n = 3 independent experimental repeats. ND = not detectable.
(TIF)

S2 Fig FLICA (16HBEo- cells). FLICA Caspase 1 activation assay in 16HBE14o- cells treated for 3 hours with media only (control), 20 μg/ml LL-37, 20 μg/ml ScrLL-37, PAO1 at 10:1 MOI, PAO1 + LL-37 or PAO1 + ScrLL-37, demonstrating synergistic activation of caspase 1 activity by PAO1 + LL-37 compared to either LL-37 alone or PAO1 alone. Data represent means +/- SEM from n = 3 independent experimental repeats, *** p < 0.001, ** p<0.01, * p<0.05, by 2-way ANOVA with Bonferonni Post-test. ns = no significant difference.
(TIF)

S3 Fig TAMRA-LL-37 and GFP-PAO1 on NHBE cells. Enlarged image of rightmost panel from timelapse series shown in Fig 5D, showing NHBE cells treated with GFP-PAO1 (green) at 10:1 MOI and 20 μg/ml TAMRA-LL-37 (red). Cell outlines from the brightfield channel have been highlighted with white dashed lines for clarity.
(TIF)

S4 Fig GFP-PAO1 colonisation (NHBE cells). Quantitation of GFP-PAO1 on NHBE cells, comparing cells entirely labeled with TAMRA-LL-37 vs cells with discrete punctate or no TAMRA-LL-37 labelling. Graph shows pixel density of GFP-PAO1 staining in the green channel (measured using Photoshop CS) divided by the cell area in pixels. Data represent means +/- SEM from n = >20 cells per condition, ** p < 0.01 by unpaired t-test.
(TIF)

S5 Fig Timelapse series–TAMRA-LL-37 and Sytox green (NHBE cells). Timelapse series of images taken by confocal microscopy showing NHBE cells pre-infected for 1 hour with PAO1 at 10:1 MOI and stained with 1 μg/ml Hoechst (blue) to label nuclei and 1 μM Sytox Green (green) to detect dead cells, followed by incubation with 20 μg/ml TAMRA-LL-37 (red). Merged and single channel (greyscale) images shown for the timepoints indicated. White arrows identify cells where TAMRA-LL-37 labelling can be seen prior to Sytox green. Sytox green is also seen to displace Hoechst staining on nuclei in those cells that have taken it up. Scale bar = 50 μm.
(TIF)

S6 Fig Effect of cathepsin B inhibition on FLICA (16HBEo- cells). FLICA Caspase 1 activation assay in 16HBE14o- cells treated for 3 hours with vehicle control (DMSO), 20 μg/ml LL-37, PAO1 at 10:1 MOI, or PAO1 + LL-37 +/- the cathepsin B inhibitor CA-074-Me (20 μM), recapitulating the CA-074-Me-mediated inhibition of caspase 1 activation by LL-37 in infected cells observed in NHBE primary cells. Data represent means +/- SEM from n = 3 independent experimental repeats, *** p < 0.001, ** p<0.01, * p < 0.05 versus PAO1 + LL-37 + CA-074-Me condition, by 2-way ANOVA with Bonferonni Post-test.
(TIF)

## References

[1] Snell N, Strachan D., Hubbard R., Gibson J., Limb E., Gupta R., Martin A., Laffan M., Jarrold I. Burden of lung disease in the UK; findings from the British Lung Foundation's 'respiratory health of the nation' project. European Respiratory Journal. 2016;48:PA4913 10.1183/13993003.congress-2016.PA4913

[2] Bals R, Wang X, Zasloff M, Wilson JM. The peptide antibiotic LL-37/hCAP-18 is expressed in epithelia of the human lung where it has broad antimicrobial activity at the airway surface. Proc Natl Acad Sci U S A. 1998;95(16):9541–6. Epub 1998/08/05. 9689116 10.1073/pnas.95.16.9541 PMC21374

[3] Hiemstra PS, Amatngalim GD, van der Does AM, Taube C. Antimicrobial Peptides and Innate Lung Defenses: Role in Infectious and Noninfectious Lung Diseases and Therapeutic Applications. Chest. 2016;149(2):545–51. Epub 2015/10/27. 10.1378/chest.15-1353 . 26502035

[4] Cox S, McHugh BJ, Davidson DJ, Dorin JR. Mammalian Antimicrobial Peptides; Defensins and Cathelicidins In: Tang YW, Sussman M, Liu D, Poxton IR, Schwartzman J, Merritt A, editors. Molecular Medical Microbiology, 2nd edition: Academic Press; 2014 p. 539–65.

[5] Gwyer Findlay E, Currie SM, Davidson DJ. Cationic host defence peptides: potential as antiviral therapeutics. BioDrugs. 2013;27(5):479–93. Epub 2013/05/08. 10.1007/s40259-013-0039-0 23649937 PMC3775153

[6] Mansour SC, Pena OM, Hancock RE. Host defense peptides: front-line immunomodulators. Trends Immunol. 2014;35(9):443–50. Epub 2014/08/13. 10.1016/j.it.2014.07.004 . 25113635

[7] Beaumont PE, Li H-N, Davidson DJ. LL-37: An Immunomodulatory Antimicrobial Host Defence Peptide. In: Hiemstra PS, Zaat SAJ, editors. Antimicrobial Peptides and Innate Immunity. Basel: Springer Basel; 2013 p. 97–121.

[8] Beaumont PE, McHugh B, Gwyer Findlay E, Mackellar A, Mackenzie KJ, Gallo RL, et al Cathelicidin host defence peptide augments clearance of pulmonary Pseudomonas aeruginosa infection by its influence on neutrophil function in vivo. PLoS One. 2014;9(6):e99029 Epub 2014/06/03. 10.1371/journal.pone.0099029 24887410 PMC4041793

[9] Currie SM, Gwyer Findlay E, McFarlane AJ, Fitch PM, Bottcher B, Colegrave N, et al Cathelicidins Have Direct Antiviral Activity against Respiratory Syncytial Virus In Vitro and Protective Function In Vivo in Mice and Humans. J Immunol. 2016;196(6):2699–710. Epub 2016/02/14. 10.4049/jimmunol.1502478 26873992 PMC4777919

[10] Kovach MA, Ballinger MN, Newstead MW, Zeng X, Bhan U, Yu FS, et al Cathelicidin-related antimicrobial peptide is required for effective lung mucosal immunity in Gram-negative bacterial pneumonia. J Immunol. 2012;189(1):304–11. Epub 2012/05/29. 10.4049/jimmunol.1103196 22634613 PMC3566644

[11] Coorens M, Banaschewski BJH, Baer BJ, Yamashita C, van Dijk A, Haagsman HP, et al Killing of P. aeruginosa by chicken cathelicidin-2 is immunogenically silent, preventing lung inflammation in vivo. Infect Immun. 2017 Epub 2017/09/28. 10.1128/IAI.00546-17 28947647 PMC5695126

[12] Semple F, Dorin JR. beta-Defensins: multifunctional modulators of infection, inflammation and more? J Innate Immun. 2012;4(4):337–48. Epub 2012/03/24. 10.1159/000336619 . 22441423 PMC6784047

[13] Hancock RE, Haney EF, Gill EE. The immunology of host defence peptides: beyond antimicrobial activity. Nature reviews Immunology. 2016;16(5):321–34. Epub 2016/04/19. 10.1038/nri.2016.29 . 27087664

[14] Broz P, Monack DM. Newly described pattern recognition receptors team up against intracellular pathogens. Nat Rev Immunol. 2013;13(8):551–65. Epub 2013/07/13. 10.1038/nri3479 . 23846113

[15] Takeuchi O, Akira S. Pattern recognition receptors and inflammation. Cell. 2010;140(6):805–20. Epub 2010/03/23. 10.1016/j.cell.2010.01.022 . 20303872

[16] Elssner A, Duncan M, Gavrilin M, Wewers MD. A novel P2X7 receptor activator, the human cathelicidin-derived peptide LL37, induces IL-1 beta processing and release. J Immunol. 2004;172(8):4987–94. Epub 2004/04/07. . 15067080 10.4049/jimmunol.172.8.4987

[17] Kahlenberg JM, Carmona-Rivera C, Smith CK, Kaplan MJ. Neutrophil extracellular trap-associated protein activation of the NLRP3 inflammasome is enhanced in lupus macrophages. J Immunol. 2013;190(3):1217–26. Epub 2012/12/26. 10.4049/jimmunol.1202388 23267025 PMC3552129

[18] Broz P, Dixit VM. Inflammasomes: mechanism of assembly, regulation and signalling. Nat Rev Immunol. 2016;16(7):407–20. 10.1038/nri.2016.58 27291964

[19] Barlow PG, Beaumont PE, Cosseau C, Mackellar A, Wilkinson TS, Hancock RE, et al The human cathelicidin LL-37 preferentially promotes apoptosis of infected airway epithelium. Am J Respir Cell Mol Biol. 2010;43(6):692–702. Epub 2010/01/26. 10.1165/rcmb.2009-0250OC 20097832 PMC2993089

[20] Saiman L, Prince A. Pseudomonas aeruginosa pili bind to asialoGM1 which is increased on the surface of cystic fibrosis epithelial cells. J Clin Invest. 1993;92(4):1875–80. Epub 1993/10/01. 10.1172/JCI116779 8104958 PMC288352

[21] Pier GB, Grout M, Zaidi TS, Olsen JC, Johnson LG, Yankaskas JR, et al Role of mutant CFTR in hypersusceptibility of cystic fibrosis patients to lung infections. Science. 1996;271(5245):64–7. . 8539601 10.1126/science.271.5245.64 PMC3677515

[22] Currie AJ, Speert DP, Davidson DJ. Pseudomonas aeruginosa: role in the pathogenesis of the CF lung lesion. Semin Respir Crit Care Med. 2003;24(6):671–80. Epub 2005/08/10. 10.1055/s-2004-815663 . 16088583

[23] Chi E, Mehl T, Nunn D, Lory S. Interaction of Pseudomonas aeruginosa with A549 pneumocyte cells. Infect Immun. 1991;59(3):822–8. Epub 1991/03/01. 1671777 10.1128/iai.59.3.822-828.1991 PMC258333

[24] Miao EA, Ernst RK, Dors M, Mao DP, Aderem A. Pseudomonas aeruginosa activates caspase 1 through Ipaf. Proc Natl Acad Sci U S A. 2008;105(7):2562–7. Epub 2008/02/08. 10.1073/pnas.0712183105 18256184 PMC2268176

[25] Sutterwala FS, Mijares LA, Li L, Ogura Y, Kazmierczak BI, Flavell RA. Immune recognition of Pseudomonas aeruginosa mediated by the IPAF/NLRC4 inflammasome. J Exp Med. 2007;204(13):3235–45. Epub 2007/12/12. 10.1084/jem.20071239 18070936 PMC2150987

[26] Miao EA, Mao DP, Yudkovsky N, Bonneau R, Lorang CG, Warren SE, et al Innate immune detection of the type III secretion apparatus through the NLRC4 inflammasome. Proc Natl Acad Sci U S A. 2010;107(7):3076–80. Epub 2010/02/06. 10.1073/pnas.0913087107 20133635 PMC2840275

[27] Deng Q, Wang Y, Zhang Y, Li M, Li D, Huang X, et al Pseudomonas aeruginosa Triggers Macrophage Autophagy To Escape Intracellular Killing by Activation of the NLRP3 Inflammasome. Infect Immun. 2016;84(1):56–66. Epub 2015/10/16. 10.1128/IAI.00945-15 26467446 PMC4694000

[28] Rimessi A, Bezzerri V, Patergnani S, Marchi S, Cabrini G, Pinton P. Mitochondrial Ca2+-dependent NLRP3 activation exacerbates the Pseudomonas aeruginosa-driven inflammatory response in cystic fibrosis. Nat Commun. 2015;6:6201 Epub 2015/02/05. 10.1038/ncomms7201 . 25648527

[29] Balakrishnan A, Karki R, Berwin B, Yamamoto M, Kanneganti TD. Guanylate binding proteins facilitate caspase-11-dependent pyroptosis in response to type 3 secretion system-negative Pseudomonas aeruginosa. Cell Death Discov. 2018;5:3 Epub 2018/08/01. 10.1038/s41420-018-0068-z 30062052 PMC6060091

[30] Tang A, Sharma A, Jen R, Hirschfeld AF, Chilvers MA, Lavoie PM, et al Inflammasome-mediated IL-1beta production in humans with cystic fibrosis. PLoS One. 2012;7(5):e37689 Epub 2012/06/01. 10.1371/journal.pone.0037689 22649552 PMC3359311

[31] Jendrossek V, Grassme H, Mueller I, Lang F, Gulbins E. Pseudomonas aeruginosa-induced apoptosis involves mitochondria and stress-activated protein kinases. Infect Immun. 2001;69(4):2675–83. Epub 2001/03/20. 10.1128/IAI.69.4.2675-2683.2001 11254634 PMC98206

[32] Pier GB, Grout M, Zaidi TS. Cystic fibrosis transmembrane conductance regulator is an epithelial cell receptor for clearance of Pseudomonas aeruginosa from the lung. Proc Natl Acad Sci U S A. 1997;94(22):12088–93. Epub 1997/10/29. 9342367 10.1073/pnas.94.22.12088 PMC23711

[33] Fleiszig SM, Zaidi TS, Fletcher EL, Preston MJ, Pier GB. Pseudomonas aeruginosa invades corneal epithelial cells during experimental infection. Infect Immun. 1994;62(8):3485–93. Epub 1994/08/01. 8039920 10.1128/iai.62.8.3485-3493.1994 PMC302982

[34] Sandgren S, Wittrup A, Cheng F, Jonsson M, Eklund E, Busch S, et al The human antimicrobial peptide LL-37 transfers extracellular DNA plasmid to the nuclear compartment of mammalian cells via lipid rafts and proteoglycan-dependent endocytosis. J Biol Chem. 2004;279(17):17951–6. Epub 2004/02/14. 10.1074/jbc.M311440200 . 14963039

[35] Henzler Wildman KA, Lee DK, Ramamoorthy A. Mechanism of Lipid Bilayer Disruption by the Human Antimicrobial Peptide, LL-37. Biochemistry. 2003;42(21):6545–58. 10.1021/bi0273563 . 12767238

[36] Gutsmann T, Larrick JW, Seydel U, Wiese A. Molecular mechanisms of interaction of rabbit CAP18 with outer membranes of gram-negative bacteria. Biochemistry. 1999;38(41):13643–53. Epub 1999/10/16. . 10521271 10.1021/bi990643v

[37] Bjorstad A, Askarieh G, Brown KL, Christenson K, Forsman H, Onnheim K, et al The host defense peptide LL-37 selectively permeabilizes apoptotic leukocytes. Antimicrob Agents Chemother. 2009;53(3):1027–38. Epub 2008/12/17. 10.1128/AAC.01310-08 19075071 PMC2650579

[38] Li HN, Barlow PG, Bylund J, Mackellar A, Bjorstad A, Conlon J, et al Secondary necrosis of apoptotic neutrophils induced by the human cathelicidin LL-37 is not proinflammatory to phagocytosing macrophages. J Leukoc Biol. 2009;86(4):891–902. Epub 2009/07/08. 10.1189/jlb.0209050 19581375 PMC2791992

[39] Lau YE, Rozek A, Scott MG, Goosney DL, Davidson DJ, Hancock RE. Interaction and cellular localization of the human host defense peptide LL-37 with lung epithelial cells. Infect Immun. 2005;73(1):583–91. Epub 2004/12/25. 10.1128/IAI.73.1.583-591.2005 15618198 PMC538997

[40] Tang X, Basavarajappa D, Haeggstrom JZ, Wan M. P2X7 Receptor Regulates Internalization of Antimicrobial Peptide LL-37 by Human Macrophages That Promotes Intracellular Pathogen Clearance. Journal of immunology. 2015;195(3):1191–201. 10.4049/jimmunol.1402845 26116509 PMC4505952

[41] Kuroda E, Ishii KJ, Uematsu S, Ohata K, Coban C, Akira S, et al Silica crystals and aluminum salts regulate the production of prostaglandin in macrophages via NALP3 inflammasome-independent mechanisms. Immunity. 2011;34(4):514–26. 10.1016/j.immuni.2011.03.019 . 21497116

[42] Hornung V, Bauernfeind F, Halle A, Samstad EO, Kono H, Rock KL, et al Silica crystals and aluminum salts activate the NALP3 inflammasome through phagosomal destabilization. Nat Immunol. 2008;9(8):847–56. Epub 2008/07/08. 10.1038/ni.1631 18604214 PMC2834784

[43] Duncan JA, Gao X, Huang MT, O'Connor BP, Thomas CE, Willingham SB, et al Neisseria gonorrhoeae activates the proteinase cathepsin B to mediate the signaling activities of the NLRP3 and ASC-containing inflammasome. Journal of immunology. 2009;182(10):6460–9. 10.4049/jimmunol.0802696 19414800 PMC2722440

[44] McAdoo MH, Dannenberg AM Jr., Hayes CJ, James SP, Sanner JH. Inhibition of cathepsin D-type proteinase of macrophages by pepstatin, a specific pepsin inhibitor, and other substances. Infect Immun. 1973;7(4):655–65. Epub 1973/04/01. 4586863 10.1128/iai.7.4.655-665.1973 PMC422739

[45] Davidson DJ, Currie A. J., Speert D. P. Pseudomonas aeruginosa infections in individuals with cystic fibrosis: North American perspective. In: Hauser A. and Rello J, editor. Severe infections caused by Pseudomonas aeruginosa. Norwell: Kluwer Academic Publishers; 2003 p. 71–89.

[46] Grassme H, Jendrossek V, Riehle A, von Kurthy G, Berger J, Schwarz H, et al Host defense against Pseudomonas aeruginosa requires ceramide-rich membrane rafts. Nat Med. 2003;9(3):322–30. Epub 2003/02/03. 10.1038/nm823 . 12563314

[47] Garcia-Medina R, Dunne WM, Singh PK, Brody SL. Pseudomonas aeruginosa acquires biofilm-like properties within airway epithelial cells. Infect Immun. 2005;73(12):8298–305. 10.1128/IAI.73.12.8298-8305.2005 16299327 PMC1307054

[48] Cannon CL, Kowalski MP, Stopak KS, Pier GB. Pseudomonas aeruginosa-Induced Apoptosis Is Defective in Respiratory Epithelial Cells Expressing Mutant Cystic Fibrosis Transmembrane Conductance Regulator. Am J Respir Cell Mol Biol. 2003;29(2):188–97. 10.1165/rcmb.4898 . 12878584

[49] Grassme H, Kirschnek S, Riethmueller J, Riehle A, von Kurthy G, Lang F, et al CD95/CD95 ligand interactions on epithelial cells in host defense to Pseudomonas aeruginosa. Science. 2000;290(5491):527–30. . 11039936 10.1126/science.290.5491.527

[50] Kowalski MP, Pier GB. Localization of cystic fibrosis transmembrane conductance regulator to lipid rafts of epithelial cells is required for Pseudomonas aeruginosa-induced cellular activation. J Immunol. 2004;172(1):418–25. . 14688350 10.4049/jimmunol.172.1.418

[51] Santana PT, Martel J, Lai H-C, Perfettini J-L, Kanellopoulos JM, Young JD, et al Is the inflammasome relevant for epithelial cell function? Microbes and Infection. 2016;18(2):93–101. Epub 2015/11/10. 10.1016/j.micinf.2015.10.007 . 26546965

[52] Knodler LA, Crowley SM, Sham HP, Yang H, Wrande M, Ma C, et al Noncanonical inflammasome activation of caspase-4/caspase-11 mediates epithelial defenses against enteric bacterial pathogens. Cell Host Microbe. 2014;16(2):249–56. Epub 2014/08/15. 10.1016/j.chom.2014.07.002 25121752 PMC4157630

[53] De Nardo D, De Nardo CM, Latz E. New insights into mechanisms controlling the NLRP3 inflammasome and its role in lung disease. Am J Pathol. 2014;184(1):42–54. Epub 2013/11/05. 10.1016/j.ajpath.2013.09.007 24183846 PMC3873477

[54] dos Santos G, Kutuzov MA, Ridge KM. The inflammasome in lung diseases. Am J Physiol Lung Cell Mol Physiol. 2012;303(8):L627–33. Epub 2012/08/21. 10.1152/ajplung.00225.2012 22904168 PMC4747913

[55] Bowdish DM, Davidson DJ, Speert DP, Hancock RE. The human cationic peptide LL-37 induces activation of the extracellular signal-regulated kinase and p38 kinase pathways in primary human monocytes. J Immunol. 2004;172(6):3758–65. Epub 2004/03/09. . 15004180 10.4049/jimmunol.172.6.3758

[56] Scott MG, Davidson DJ, Gold MR, Bowdish D, Hancock RE. The human antimicrobial peptide LL-37 is a multifunctional modulator of innate immune responses. J Immunol. 2002;169(7):3883–91. Epub 2002/09/24. . 12244186 10.4049/jimmunol.169.7.3883

[57] Yu J, Mookherjee N, Wee K, Bowdish DM, Pistolic J, Li Y, et al Host defense peptide LL-37, in synergy with inflammatory mediator IL-1beta, augments immune responses by multiple pathways. J Immunol. 2007;179(11):7684–91. Epub 2007/11/21. . 18025214 10.4049/jimmunol.179.11.7684

[58] Schaller-Bals S, Schulze A, Bals R. Increased Levels of Antimicrobial Peptides in Tracheal Aspirates of Newborn Infants during Infection. Am J Respir Crit Care Med. 2002;165(7):992–5. 10.1164/ajrccm.165.7.200110-020 . 11934727

[59] Barlow PG, Li Y, Wilkinson TS, Bowdish DM, Lau YE, Cosseau C, et al The human cationic host defense peptide LL-37 mediates contrasting effects on apoptotic pathways in different primary cells of the innate immune system. J Leukoc Biol. 2006;80(3):509–20. Epub 2006/06/24. 10.1189/jlb.1005560 16793910 PMC1851551

[60] Solle M, Labasi J, Perregaux DG, Stam E, Petrushova N, Koller BH, et al Altered cytokine production in mice lacking P2X(7) receptors. The Journal of biological chemistry. 2001;276(1):125–32. Epub 2000/10/04. 10.1074/jbc.M006781200 . 11016935

[61] Lopez-Castejon G, Luheshi NM, Compan V, High S, Whitehead RC, Flitsch S, et al Deubiquitinases regulate the activity of caspase-1 and interleukin-1beta secretion via assembly of the inflammasome. The Journal of biological chemistry. 2013;288(4):2721–33. Epub 2012/12/05. 10.1074/jbc.M112.422238 23209292 PMC3554938

[62] Ran FA, Hsu PD, Wright J, Agarwala V, Scott DA, Zhang F. Genome engineering using the CRISPR-Cas9 system. Nature protocols. 2013;8(11):2281–308. 10.1038/nprot.2013.143 24157548 PMC3969860

