# Supplementary figures and images for: Cathelicidin is a “fire alarm”, generating protective NLRP3-dependent airway epithelial cell inflammatory responses during infection with Pseudomonas aeruginosa
Source: PLoS Pathog. 2019 Apr 12;15(4):e1007694. doi: 10.1371/journal.ppat.1007694 (PMC6481867; doi:10.1371/journal.ppat.1007694)

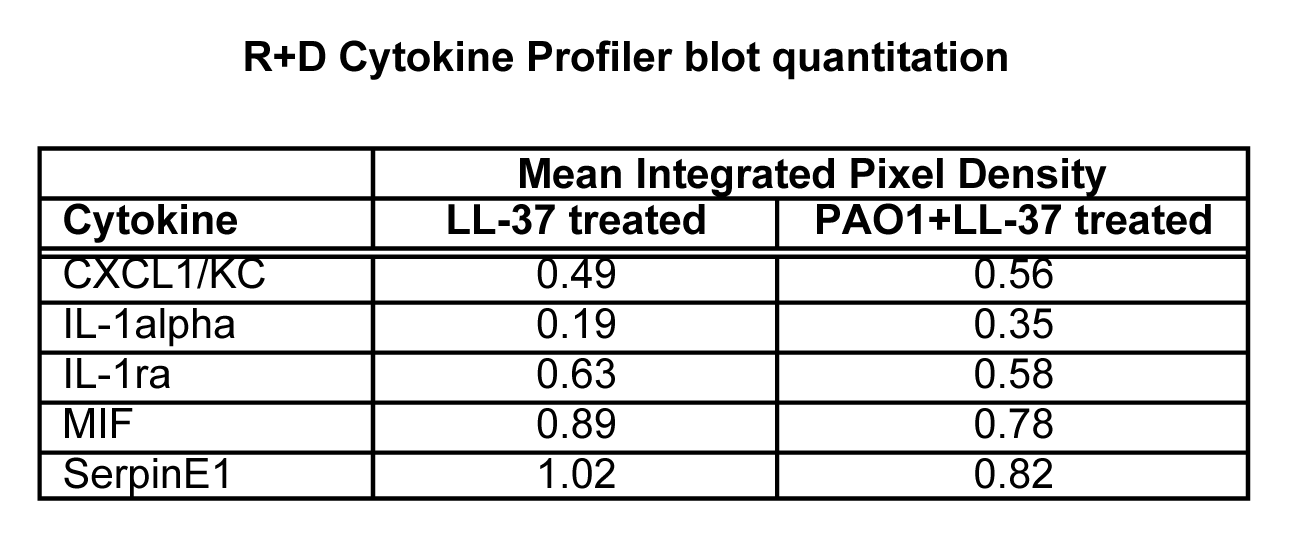

Supplement: S1 Table — Quantitation of additional cytokines released from NHBE cells after treatment. A Cytokine Profiler dot blot (R+D Systems) was probed with filtered supernatant from NHBE cells treated for 3 hours with either 20 μg/ml LL-37 or 20 μg/ml LL-37 + PAO1 at 10:1 MOI. Quantitation was performed by measuring Integrated Pixel Density of each cytokine spot in ImageJ. S1 Table lists the mean Integrated Pixel Density values for 4 measurements from each cytokine in each condition. (TIF) [file ppat.1007694.s001.tif]

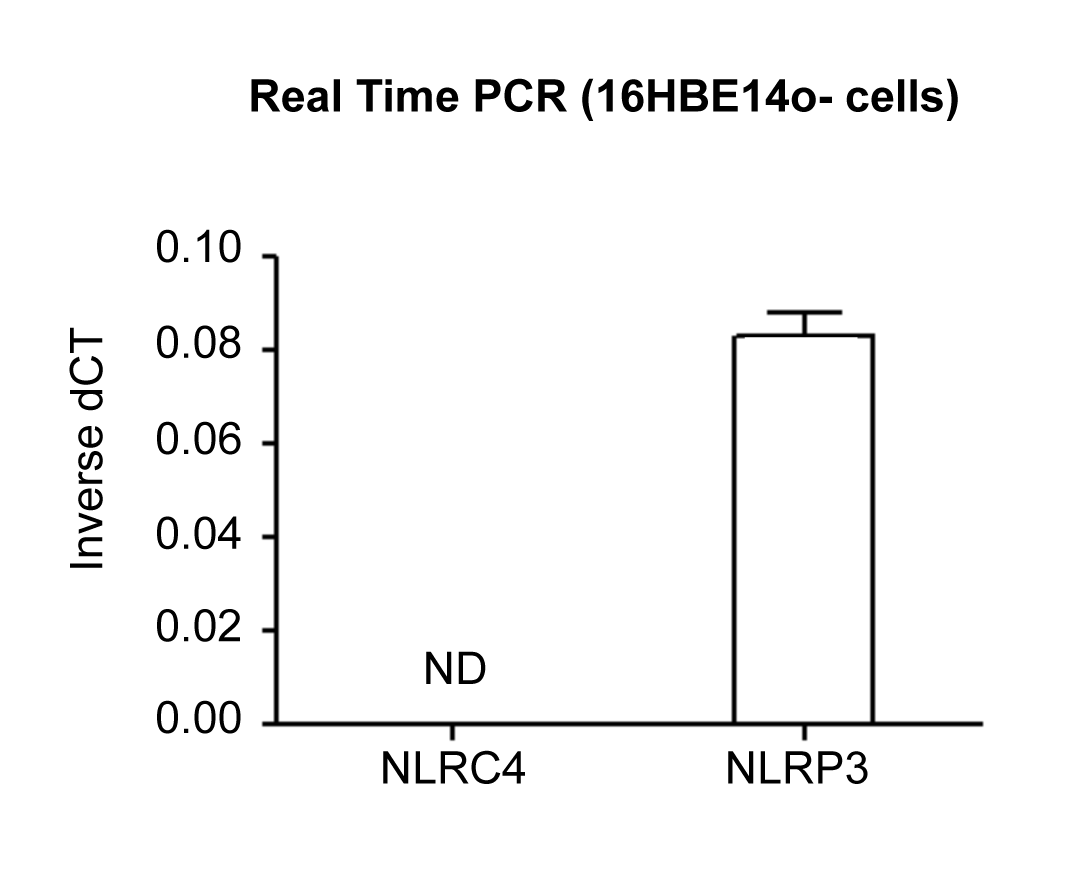

Supplement: S1 Fig — Quantitative Real Time PCR of inflammasome components NLRC4 and NLRCP3 in 16HBE14o- cells, showing no detectable NLRC4. Data represent means +/- SEM from n = 3 independent experimental repeats. ND = not detectable. (TIF) [file ppat.1007694.s002.tif]

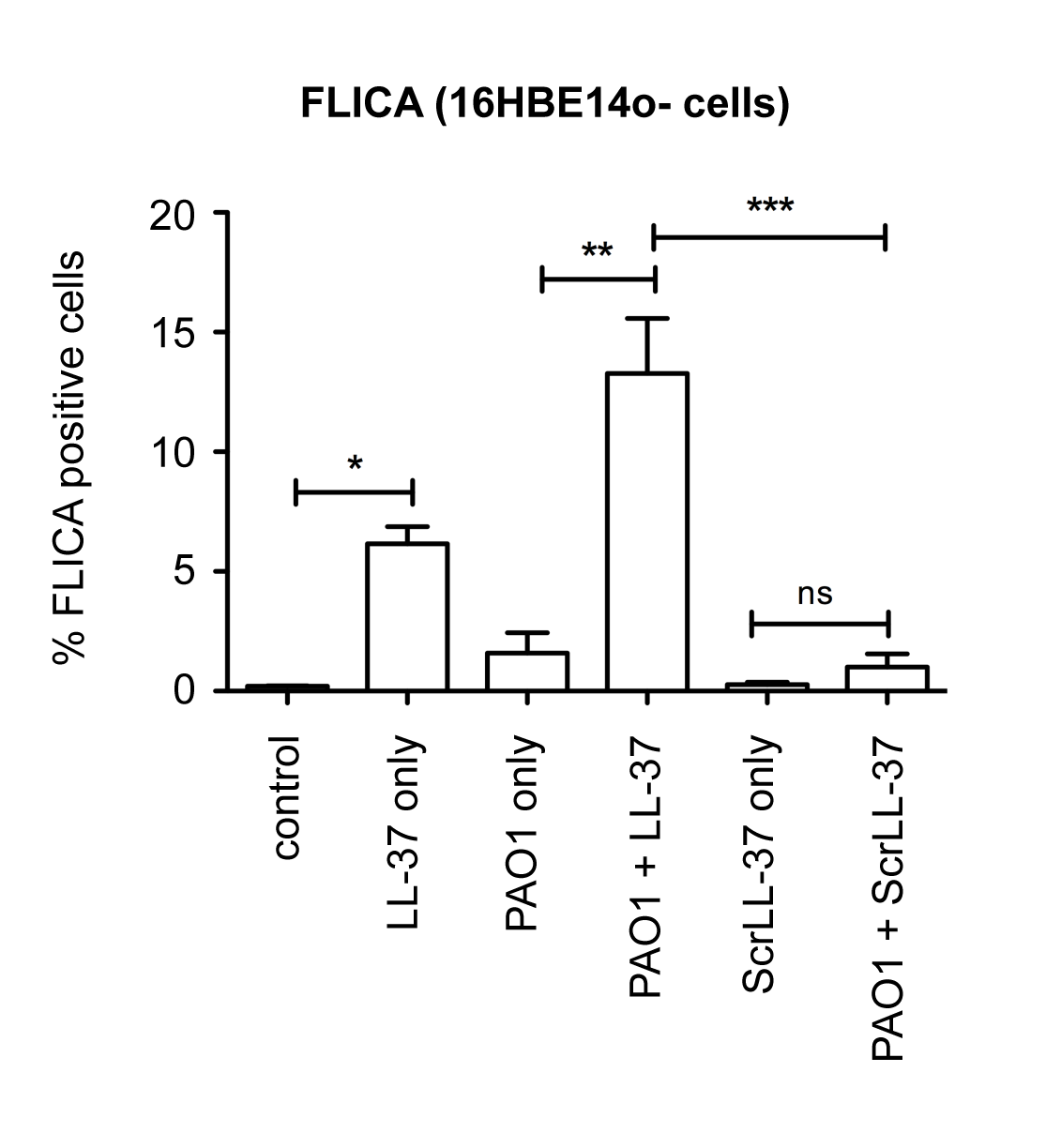

Supplement: S2 Fig — FLICA Caspase 1 activation assay in 16HBE14o- cells treated for 3 hours with media only (control), 20 μg/ml LL-37, 20 μg/ml ScrLL-37, PAO1 at 10:1 MOI, PAO1 + LL-37 or PAO1 + ScrLL-37, demonstrating synergistic activation of caspase 1 activity by PAO1 + LL-37 compared to either LL-37 alone or PAO1 alone. Data represent means +/- SEM from n = 3 independent experimental repeats, *** p < 0.001, ** p<0.01, * p<0.05, by 2-way ANOVA with Bonferonni Post-test. ns = no significant difference. (TIF) [file ppat.1007694.s003.tif]

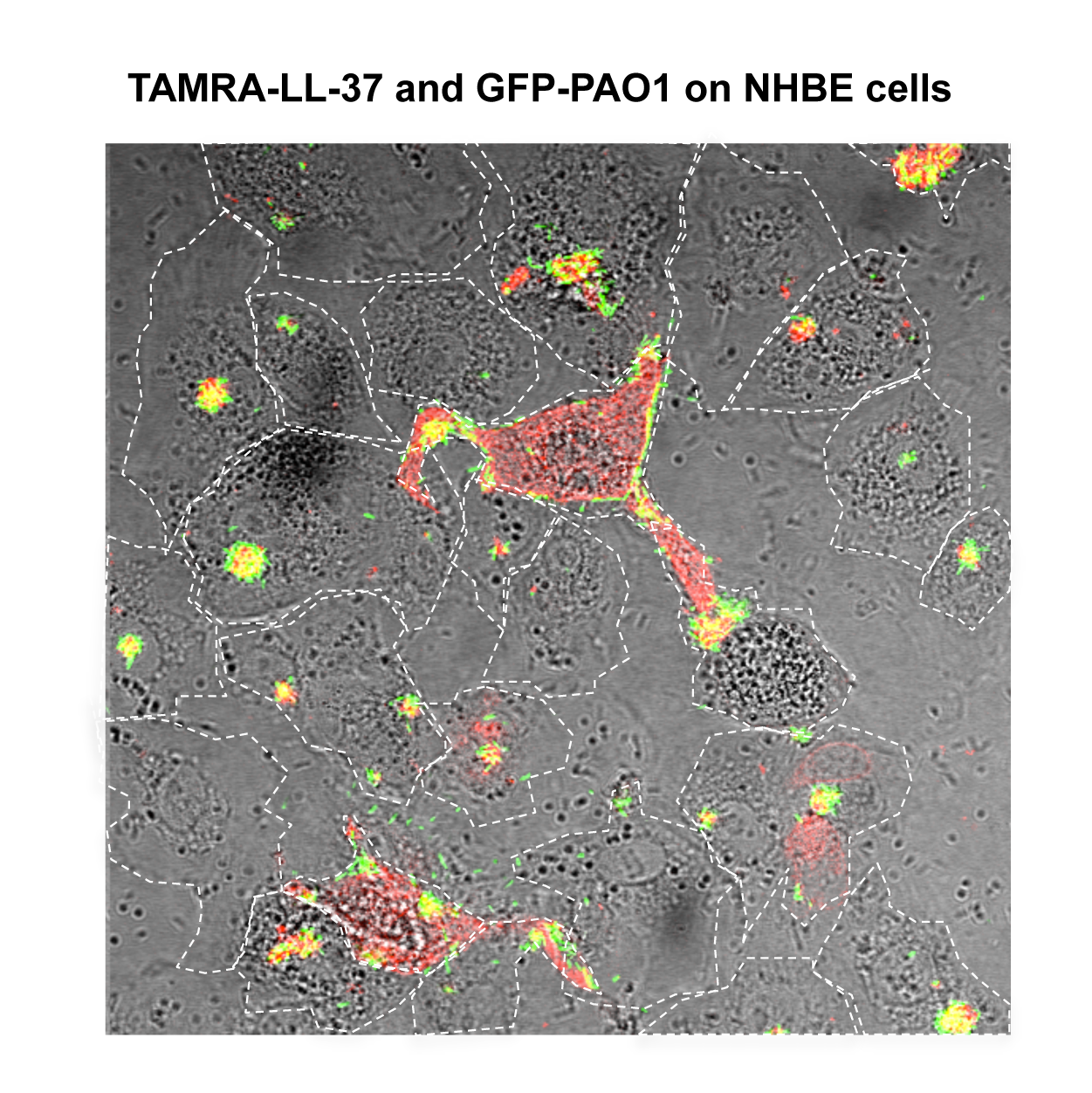

Supplement: S3 Fig — Enlarged image of rightmost panel from timelapse series shown in Fig 5D, showing NHBE cells treated with GFP-PAO1 (green) at 10:1 MOI and 20 μg/ml TAMRA-LL-37 (red). Cell outlines from the brightfield channel have been highlighted with white dashed lines for clarity. (TIF) [file ppat.1007694.s004.tif]

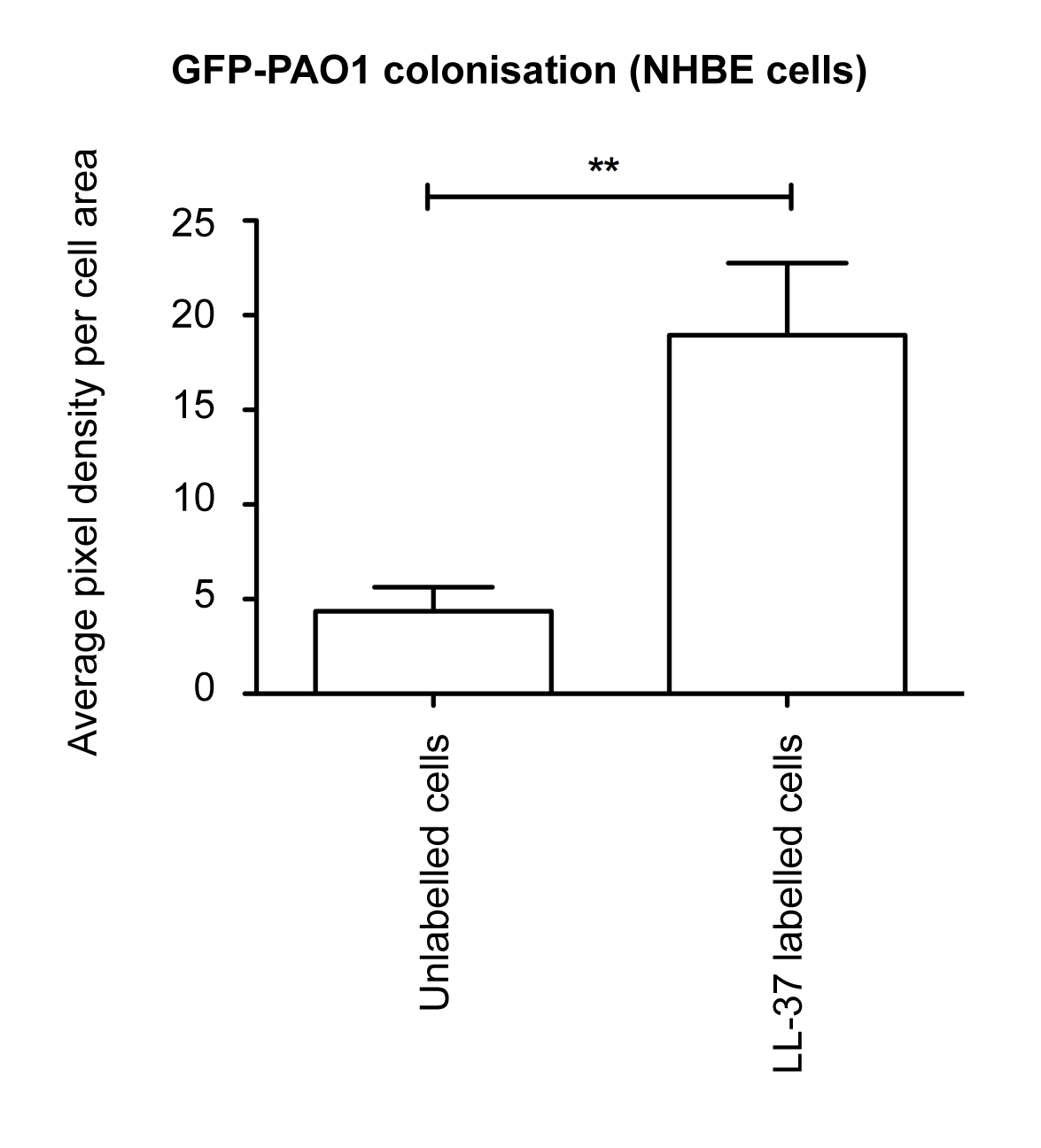

Supplement: S4 Fig — Quantitation of GFP-PAO1 on NHBE cells, comparing cells entirely labeled with TAMRA-LL-37 vs cells with discrete punctate or no TAMRA-LL-37 labelling. Graph shows pixel density of GFP-PAO1 staining in the green channel (measured using Photoshop CS) divided by the cell area in pixels. Data represent means +/- SEM from n = >20 cells per condition, ** p < 0.01 by unpaired t-test. (TIF) [file ppat.1007694.s005.tif]

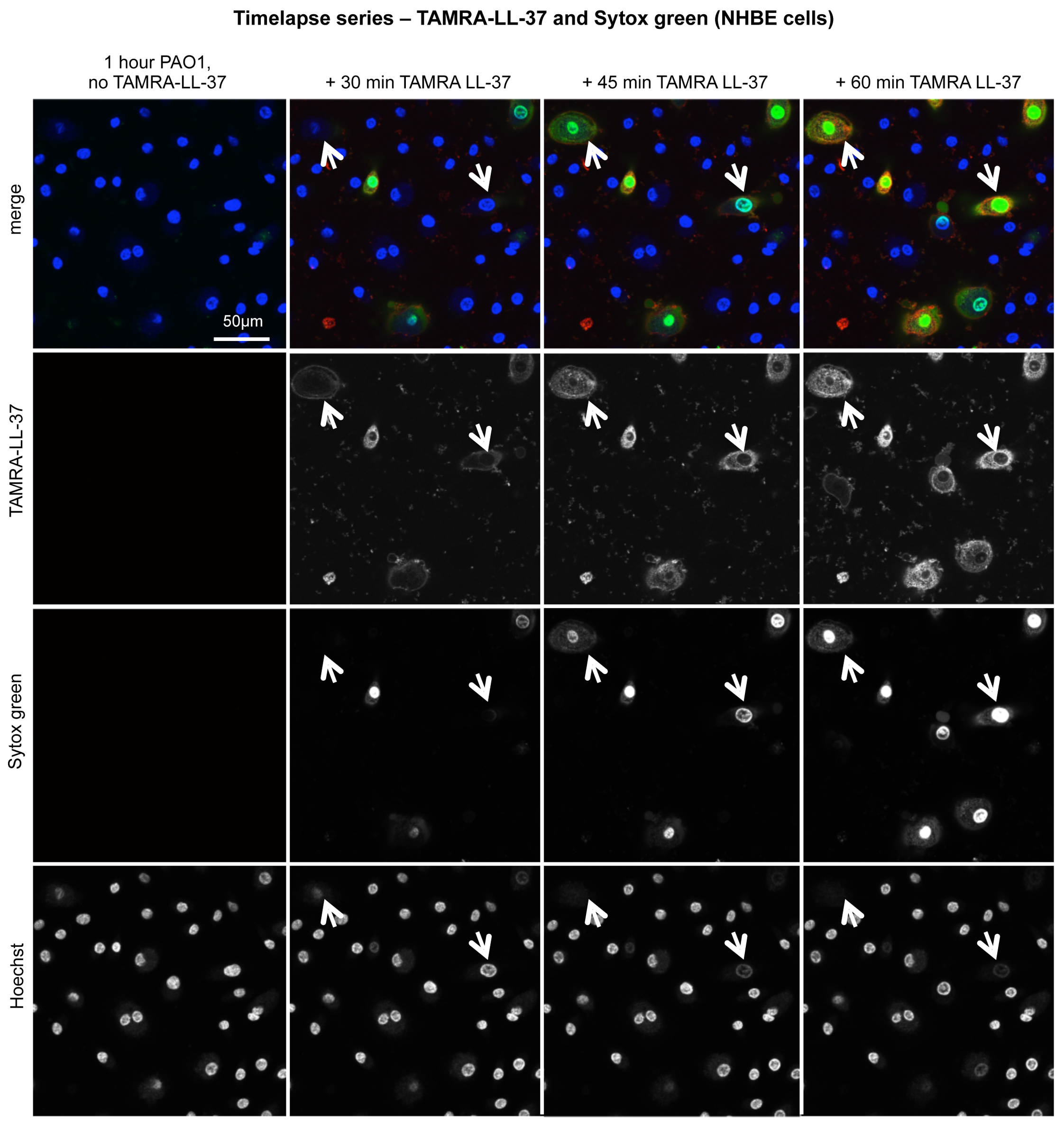

Supplement: S5 Fig — Timelapse series of images taken by confocal microscopy showing NHBE cells pre-infected for 1 hour with PAO1 at 10:1 MOI and stained with 1 μg/ml Hoechst (blue) to label nuclei and 1 μM Sytox Green (green) to detect dead cells, followed by incubation with 20 μg/ml TAMRA-LL-37 (red). Merged and single channel (greyscale) images shown for the timepoints indicated. White arrows identify cells where TAMRA-LL-37 labelling can be seen prior to Sytox green. Sytox green is also seen to displace Hoechst staining on nuclei in those cells that have taken it up. Scale bar = 50 μm. (TIF) [file ppat.1007694.s006.tif]

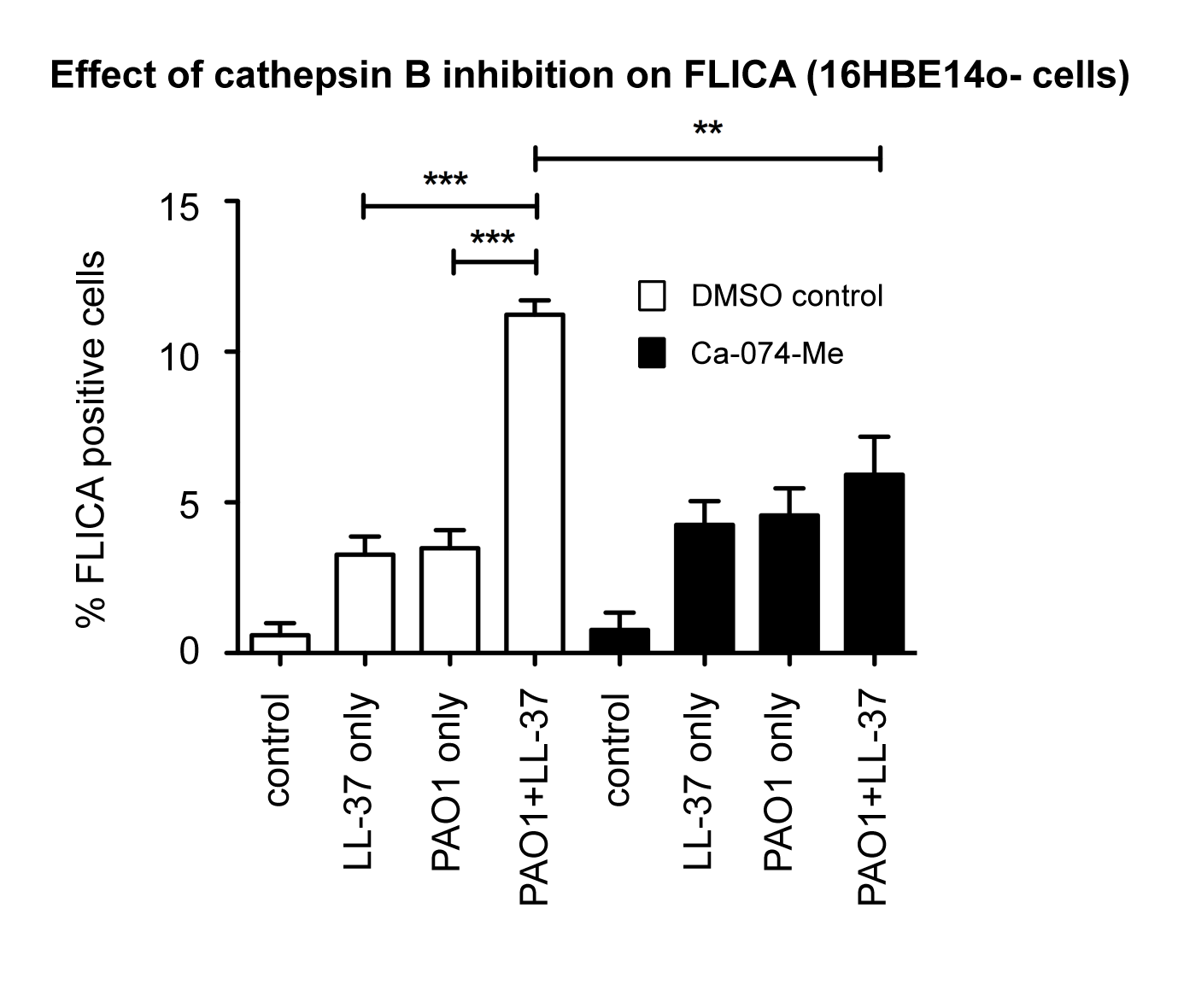

Supplement: S6 Fig — FLICA Caspase 1 activation assay in 16HBE14o- cells treated for 3 hours with vehicle control (DMSO), 20 μg/ml LL-37, PAO1 at 10:1 MOI, or PAO1 + LL-37 +/- the cathepsin B inhibitor CA-074-Me (20 μM), recapitulating the CA-074-Me-mediated inhibition of caspase 1 activation by LL-37 in infected cells observed in NHBE primary cells. Data represent means +/- SEM from n = 3 independent experimental repeats, *** p < 0.001, ** p<0.01, * p < 0.05 versus PAO1 + LL-37 + CA-074-Me condition, by 2-way ANOVA with Bonferonni Post-test. (TIF) [file ppat.1007694.s007.tif]
